# Construction of an artificial system for ambrein biosynthesis and investigation of some biological activities of ambrein

**DOI:** 10.1038/s41598-020-76624-y

**Published:** 2020-11-12

**Authors:** Yota Yamabe, Yukina Kawagoe, Kotone Okuno, Mao Inoue, Kanako Chikaoka, Daijiro Ueda, Yuko Tajima, Tadasu K. Yamada, Yoshito Kakihara, Takashi Hara, Tsutomu Sato

**Affiliations:** 1 Department of Agriculture, Faculty of Agriculture, and Graduate School of Science and Technology, Niigata University, 8050, Ikarashi-2, Nishi-ku, Niigata, Japan; 2 Department of Zoology, National Museum of Nature and Science, 4-1-1Amakubo, Tsukuba, Ibaraki Japan; 3 Division of Dental Pharmacology, Department of Tissue Regeneration and Reconstruction, Faculty of Dentistry, Niigata University Graduate School of Medical and Dental Sciences, Chuo-ku, Niigata, 951-8514 Japan

**Keywords:** Biochemistry, Chemical biology

## Abstract

Ambergris, a sperm whale metabolite, has long been used as a fragrance and traditional medication, but it is now rarely available. The odor components of ambergris result from the photooxidative degradation of the major component, ambrein. The pharmacological activities of ambergris have also been attributed to ambrein. However, efficient production of ambrein and odor compounds has not been achieved. Here, we constructed a system for the synthesis of ambrein and odor components. First, we created a new triterpene synthase, “ambrein synthase,” for mass production of ambrein by redesigning a bacterial enzyme. The ambrein yields were approximately 20 times greater than those reported previously. Next, an efficient photooxidative conversion system from ambrein to a range of volatiles of ambergris was established. The yield of volatiles was 8–15%. Finally, two biological activities, promotion of osteoclast differentiation and prevention of amyloid β-induced apoptosis, were discovered using the synthesized ambrein.

## Introduction

**Figure 1:**
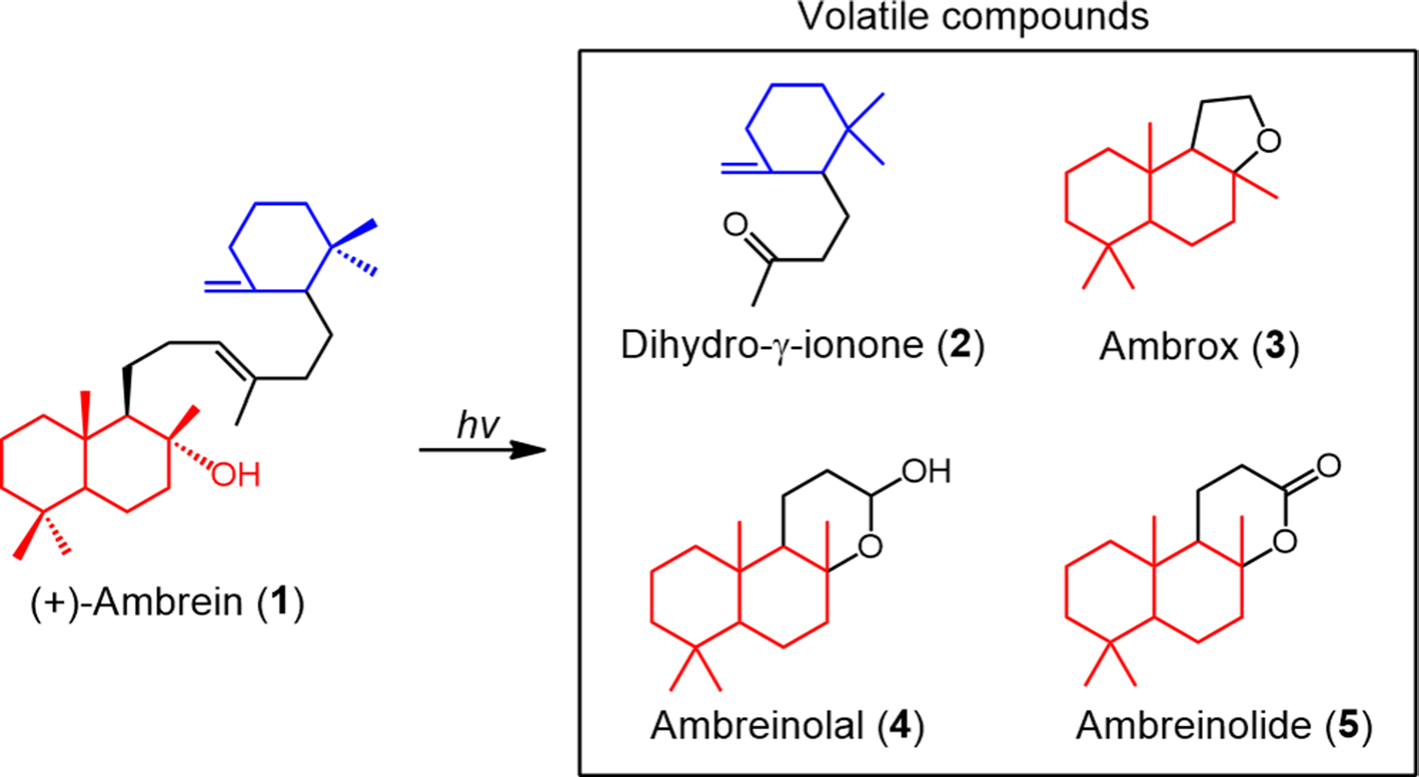
Photooxygenation of **1** to produce volatile compounds (**2**–**5**). Tricyclic triterpene, (+)-ambrein (**1**), is the primary source of ambergris-derived odor components^2–4,9–11^. Photooxidative degradation of **1** by^1^O_2_ around its central double bond produces volatile compounds with smaller molecular weights^2–4,9–11^. **2-5** are the main components identified. Tricyclic volatiles **3**–**5** originate from the bicyclic part of **1**, while the smaller fragment **2** is related to the monocyclic part of **1**^2–4,9–11^.

Ambergris, a metabolic product of the sperm whale (*Physeter catodon* or *Physeter macrocephalus*), accumulates as gut concretions with a probability of about 1%, and is one of the most valuable scents of animal origin^1–3^. Ambergris, which exhibits various medicinal properties, has also been used worldwide as a traditional medication for maladies such as migraines, the common cold, constipation, disease of the nervous system, and rheumatism^4–6^. In addition, it is often used as an aphrodisiac^7^. However, ambergris is almost inaccessible because sperm whales are now protected under the Convention on the International Trade of Endangered Species of Wild Fauna and Flora. On rare occasions, jetsam ambergris is found on beaches around the world, and traded at a high price^8^. The odor components of ambergris result from the photooxidative degradation of the major component, ambrein (**1**)^2,3,9–11^, and the pharmacological activity is also believed to be due to **1** (Fig. 1)^5,6^. To date, efficient conversion to both **1** and odor components (**2**–**5**, Fig. 1) has never been achieved. The reported yields of chemical synthesis of **1** and conversion of **1** to odor components are 1.3–3.8% and approximately 1%, respectively^11–14^. In addition, since the biosynthetic pathway of **1** in sperm whales remains unclear, it is not possible to utilize the biosynthetic enzyme that produces **1**.

**Figure 2:**
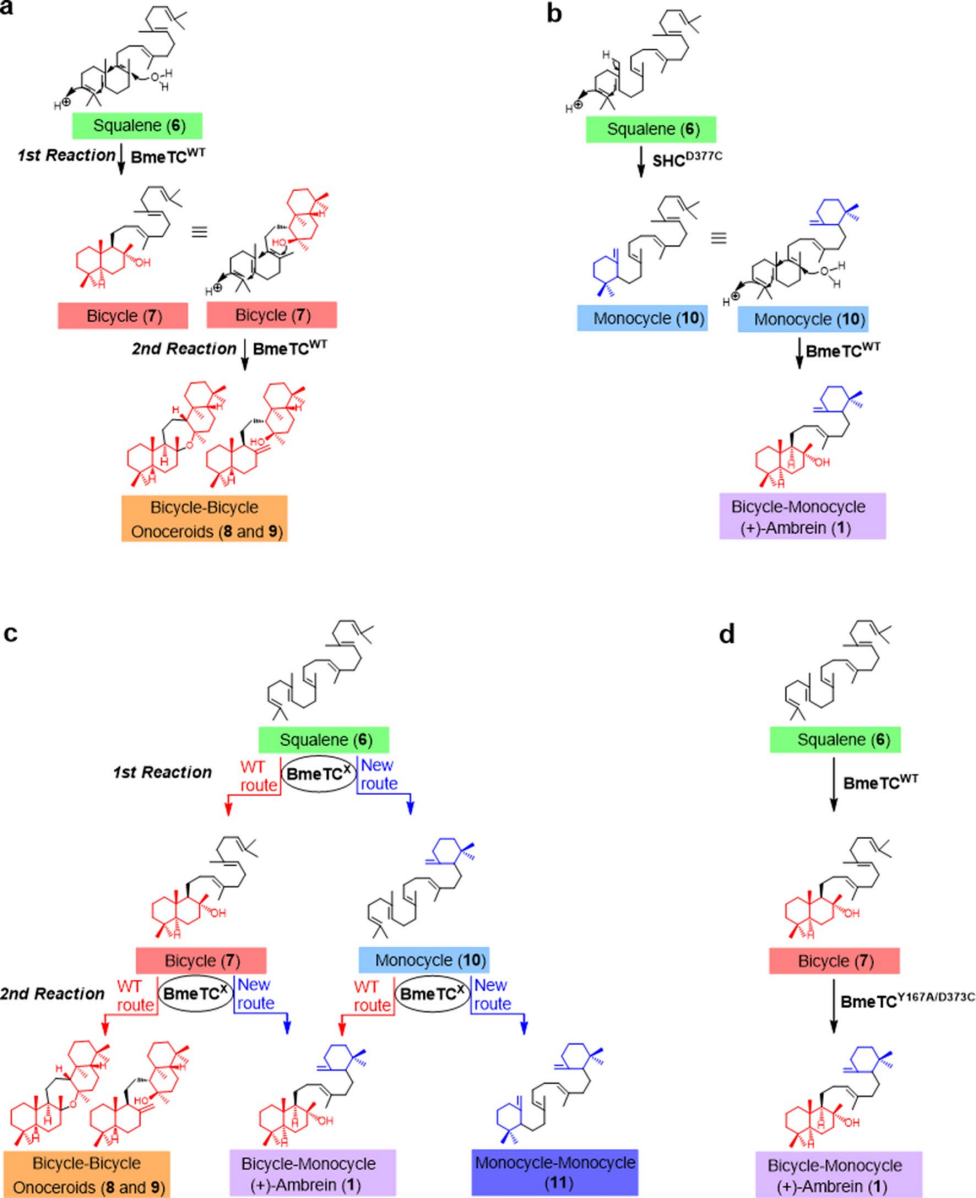
The enzymatic reaction pathway described in this study. (**a**) Mechanism of conversion of **6** to **8** and **9** by BmeTC^WT^. (**b**) Mechanism of conversion of **6** to **1** by SHC^D377C^ and BmeTC^WT^. (**c**) Reaction pathway by BmeTC^D373C^ or BmeTC^Y167A/D373C^. BmeTC^D373C^ or BmeTC^Y167A/D373C^ form final products (**1**, **8**, **9** and **11**) through a two-step reaction from **6** via intermediates (**7** and **10**) and may have catalytic functions in two pathways (WT and New route). (**d**) Two enzyme system (BmeTC^WT^ and BmeTC^Y167A/D373C^) constructed in this study.

Successful artificial enzymatic synthesis of **1** was reported in 2013 using onoceroid synthase, BmeTC, from *Bacillus megaterium,* which sequentially cyclizes both termini of squalene (**6**; Fig. 2a)^12^. Presumably, BmeTC first cyclizes one side end of **6,** converts it into a bicycle **7**, and then incorporates **7** into the same active site as a substrate and cyclizes the remaining terminal to produce onoceroids **8** and **9** (Fig. 2a). BmeTC catalyzes this two-step reaction (acycle [**6**] → bicycle [**7**] → bicycle-bicycle [**8** and **9**]; Fig. 2a)^15^. BmeTC can successfully form **1** using an abnormal monocyclic product (**10**), synthesized by a squalene-hopene cyclase variant (SHC^D377C^), as a substrate (Fig. 2b)^15^. Hence, enzymatic synthesis of **1** from inexpensive **6** was achieved via the route “acycle **6** → monocycle **10** → bicycle-monocycle **1**” (Fig. 2b)^15^. Furthermore, the BmeTC variant BmeTC^D373C^, which is an equivalent point mutation as SHC^D377C^, could reportedly synthesize **1** from **6** both in vitro and in vivo (Fig. 2c)^16–18^. By acquiring a “new route” that forms a monocycle **10**, in addition to the wild-type route (WT route) that forms bicycle **7**, BmeTC^D373C^ synthesizes **1** via the two pathways, **6** → **7** → **1** (WT route → new route) and **6** → **10** → **1** (new route → WT route) (Fig. 2c). In a bioreactor, the production of **1** by BmeTC^D373C^ in yeast *Pichia pastoris* reached a maximum titer of 105 mg L^-1^ of culture medium^17^, which was higher than that produced using other host systems or with the two enzyme system involving SHC^D377C^ and BmeTC^WT^ (Supplementary Table 1)^17–19^.

Since the production efficiency of **1** and odor components was still too low for industrialization, we sought to construct an artificial synthesis system that is dramatically more efficient than the one used currently. First, we created an “ambrein (**1**) synthase” that produces a higher yield of **1** than that of the final products (**8** and **9**) produced by wild-type BmeTC. Next, we established an efficient photooxidative conversion system to transform **1** to volatile components of ambergris. Finally, the synthesized **1** was analyzed for its bioactivities that have not yet been explored, by using cell culture assays. In this study, we focused on the effects of **1** on bone cell differentiation and against amyloid β neurotoxicity, since ambergris has been used in traditional medicine to treat rheumatism and disease of the nervous system.

## Results

### Screening for variants suitable for the synthesis of 1

**Figure 3:**
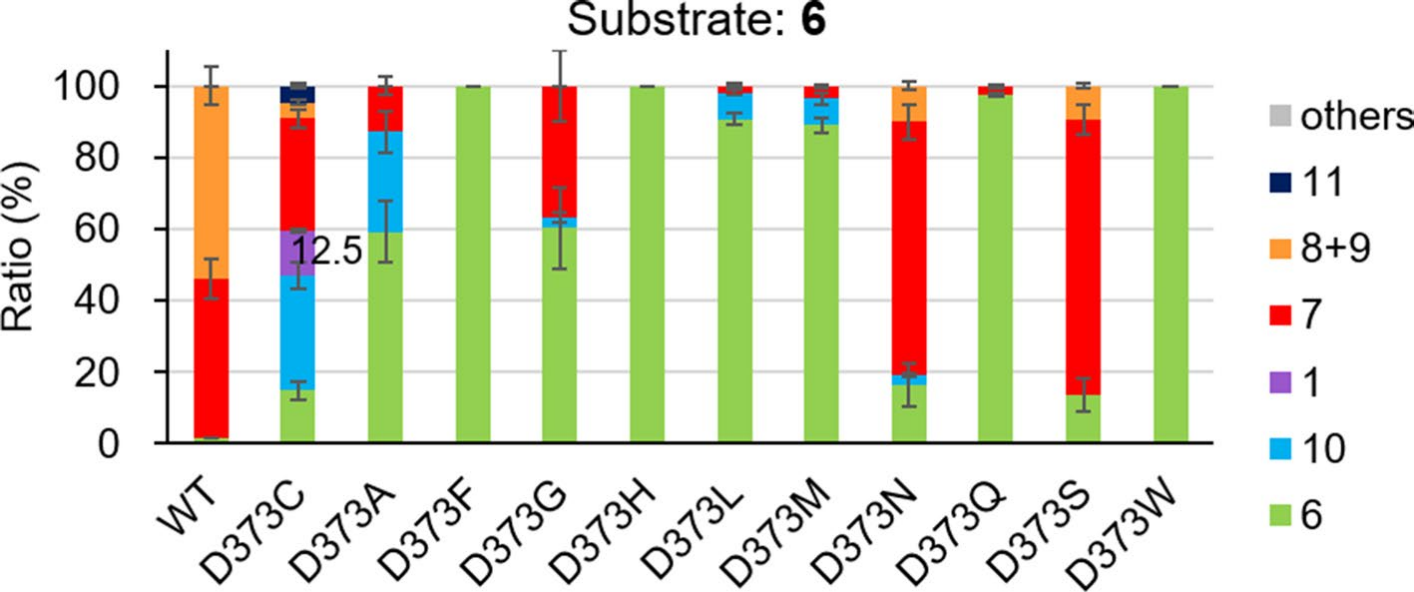
Yield of products produced by BmeTC^X^ from substrate **6** in a cell-free system.

**Figure 4:**
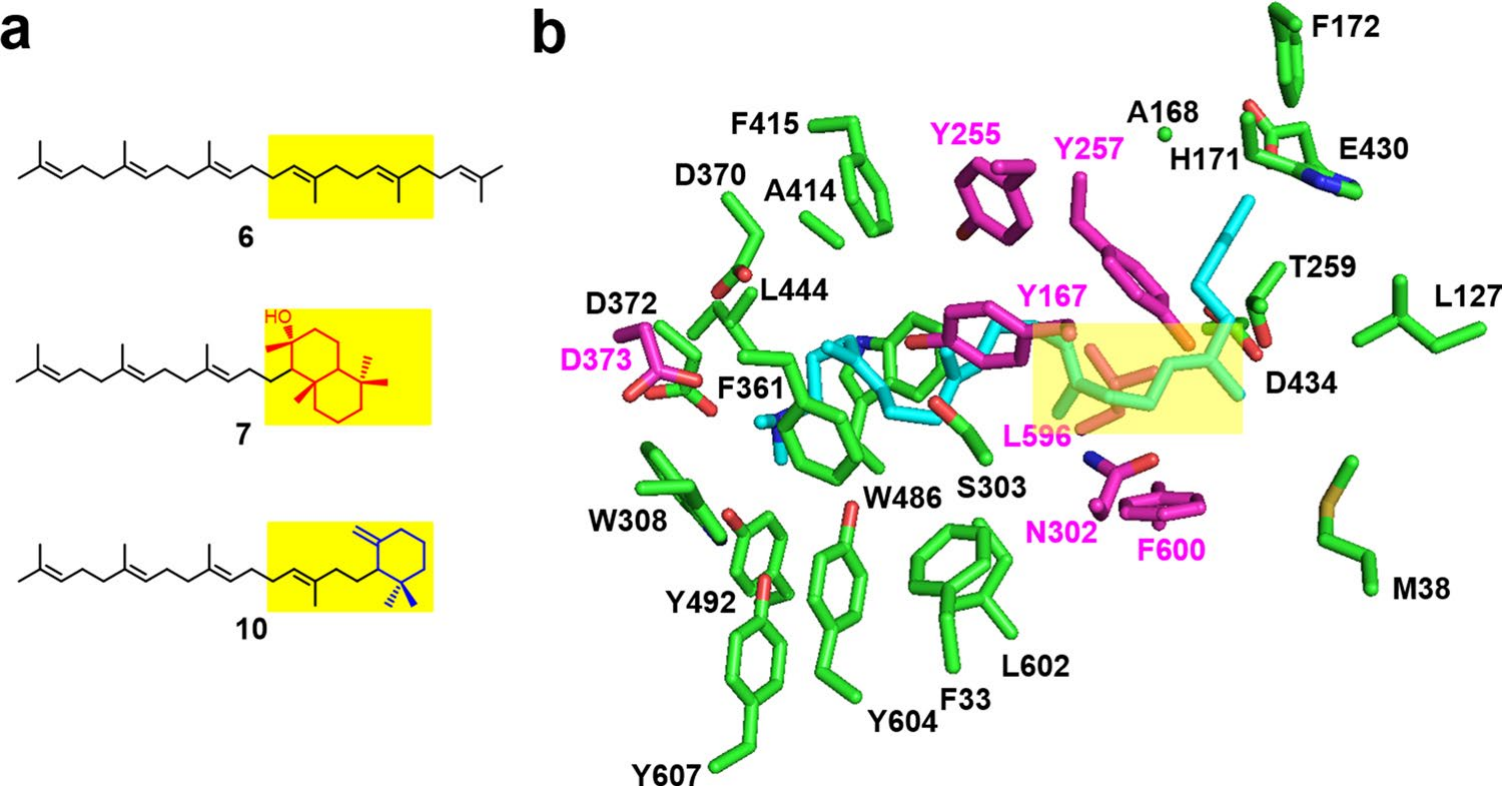
Selection of residues, presumably located near a bicyclic or monocyclic structure, during the second step of the reaction. (**a**) Structures of substrates **6**, **7**, and **10**. Positions corresponding to bicyclic or monocyclic structure on the substrates are shown in yellow. (**b**) Homology model of BmeTC. The 3D structure of BmeTC was modeled using SWISS-MODEL. The structure was constructed using the coordinates of SHC complexed with 2-azasqualene inhibitor [Protein Data Bank (PDB) code: 1UMP] as a template and displayed using PYMOL. Blue: 2-azasqualene; pink and green: residues within 4 Å of 2-azasqualene; pink: residues targeted in this study; yellow: positions corresponding to bicyclic or monocyclic structure on the substrates.

Mutation analysis of BmeTC and SHC suggested that the D373 mutation of BmeTC was important for the synthesis of **1**. However, none of the studies have analyzed D373 variants other than BmeTC^D373C^. In this study, an *Escherichia coli* cell-free system expressing the D373 variants (Supplementary Fig. 1) substituted with 11 amino acids (C, A, F, G, H, L, M, N, Q, S and W) was used to confirm their ability to convert **6** to **1**. The results indicated that only the C mutant produced **1** (12.5% yield) (Fig. 3). This revealed that cysteine at position D373 is critical to form **1**. BmeTC^D373C^ accumulated large amounts of intermediates, bicycle **7** and monocycle **10**, during the reaction with **6** (Fig. 3), showing that the bulky bicyclic and monocyclic structures of intermediates (Fig. 4a) interfered in the second-step reaction.

**Figure 5:**
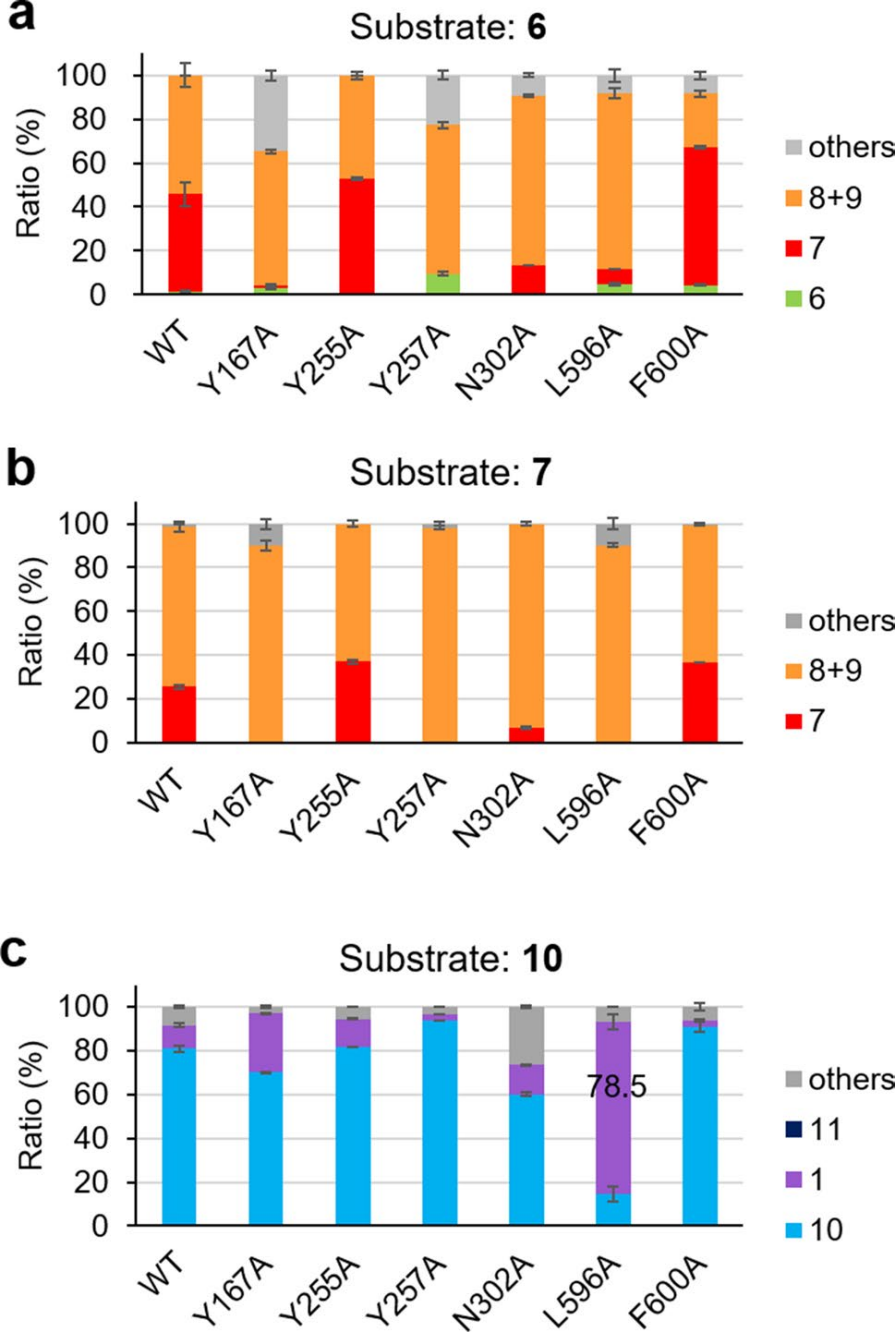
Yield of products produced by BmeTC^X^ from substrates (**6**, **7**, and **10**) in a cell-free system.

Based on this working hypothesis, 6 residues (Y167, Y255, Y257, N302, L596, and F600) that were presumed to be located near the bicyclic or monocyclic structure during the second reaction were selected based on the modeling structure of BmeTC (Fig. 4) and replaced by a smaller Ala. Enzymatic reactions of 6 Ala mutants were performed using a cell-free system (Supplementary Fig. 1). Y167A, Y257A, and N302A variants showed a higher level of activity on substrates **6** and **7** to produce **8** and **9,** compared with BmeTC^WT^ (Fig. 5a,b and Supplementary Figs. 2 and 3). Whereas the product of BmeTC^Y167A^ containing a novel tricyclic compound was previously reported^20^, the enzyme activity was first revealed in this study.

On the other hand, the yield of **1** synthesized from **10** by the L596A variant was 8 times that synthesized by BmeTC^WT^ (Fig. 5c and Supplementary Fig. 4). Thus, it may be useful to replace BmeTC^WT^ with BmeTC^L596^^A^ during the second step of the two-enzyme system consisting of SHC^D377C^ and BmeTC^WT^ (Fig. 2b). However, as the single enzyme system of BmeTC^D373C^ has been shown to be more advantageous for producing of **1** in yeast (Supplementary Table 1)^17,18^, the present study further improved upon BmeTC^D373C^.

### Construction of the system for artificial biosynthesis of 1

**Figure 6:**
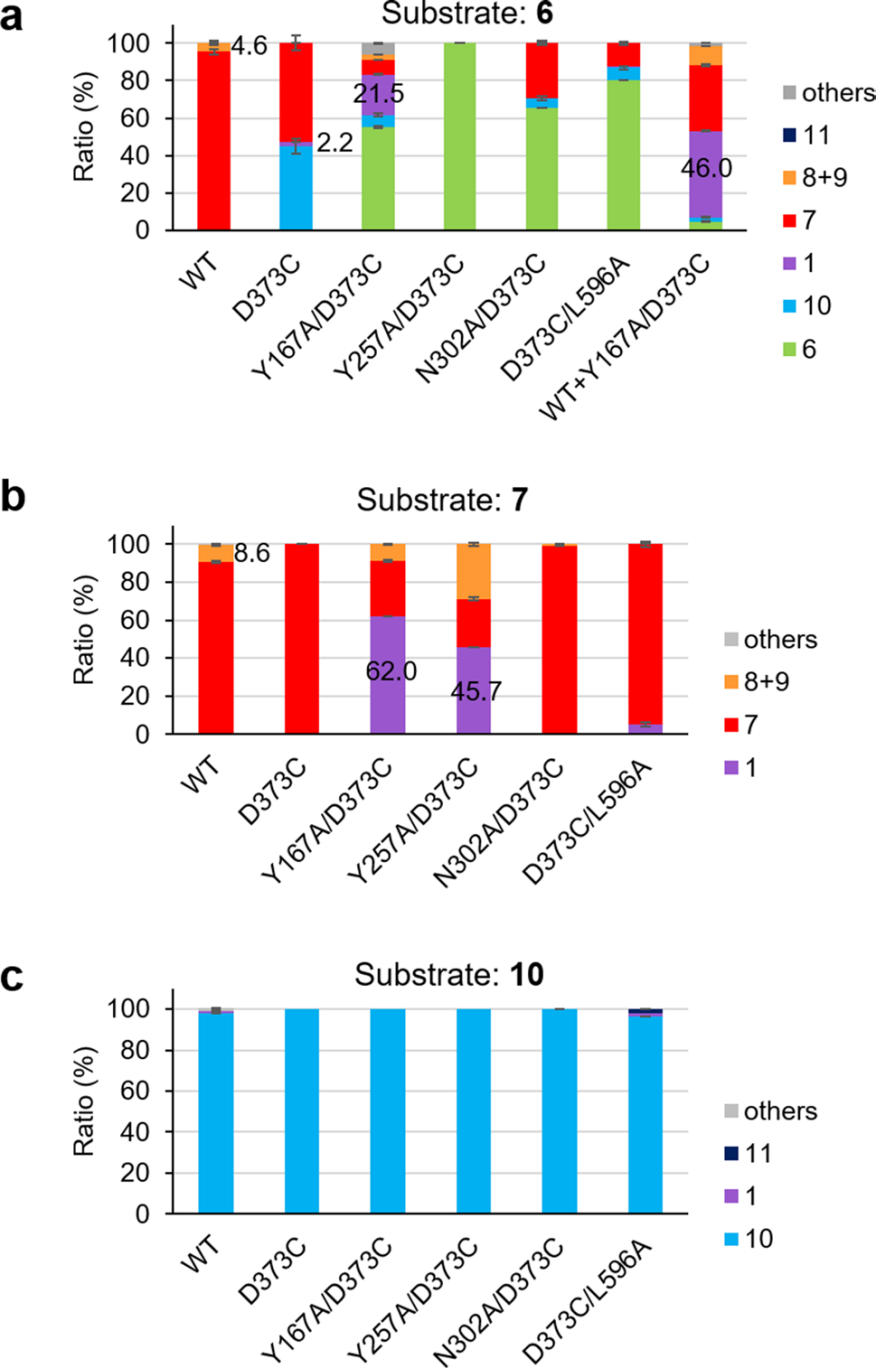
Yield of products produced by BmeTC^X^ from substrates (**6**, **7**, and **10**) in the system using purified enzymes.

In order to improve its reactivity with monocyclic **10** and bicyclic substrate **7**, 4 mutations (Y167A, Y257A, N302A, and L596A) were introduced into BmeTC^D373C^, following which the reactivity of these variants (Y167A/D373C, Y257A/D373C, N302A/D373C, and D373C/L596A) with 3 substrates (**6**, **7,** and **10**) was accurately analyzed using the purified enzyme (Fig. 6, Supplementary Figs. 5 and 6). The results indicated that of the double mutants, only BmeTC^Y167A/D373C^ produced **1** from **6**, in which the yield of **1** (21.5%) was improved approximately tenfold over that of BmeTC^D373C^ (2.2%) (Fig. 6a). If BmeTC^Y167A/D373C^ produces **1** in *P. pastoris* with the same efficacy as BmeTC^D373C 17^, an end yield of approximately 1 g **1** / L culture medium can be expected.

The conversion of **6** and **7** by BmeTC^Y167A/D373C^ to yield **1** (21.5 and 62.0%, respectively) were 4.7 and 7.2 times greater than the conversion of **6** and **7** by BmeTC^WT^ to **8** and **9** (4.6 and 8.6%, respectively) by BmeTC^WT^ (Fig. 6a,b). This indicated that the double mutant enzyme displayed activity beyond its original function to form onoceroids (**8** and **9**). Since the activity of previously constructed BmeTC^D373C^ on substrates **6** and **7** (producing 2.2 and 0% of **1**, respectively) was lower than that of BmeTC^WT^ (Fig. 6a,b), we named the novel enzyme created in this study, BmeTC^Y167A/D373C^, as “ambrein synthase.”

BmeTC^Y167A/D373C^ reacted best with **7** rather than **6** (Fig. 6a,b), indicating that the reaction with **6** was slower in the first step (**6** → **7**) than in the second step (**7** → **1**) (Fig. 2c); thus, the first step would be rate limiting. In addition, BmeTC^Y167A/D373C^ did not react with **10** (Fig. 6c), suggesting that **1** was specifically synthesized from **7** when **6** was used as a substrate, and that BmeTC^Y167A/D373C^ mainly catalyzed the reaction **6** → **7** → **1** (Fig. 2c). Hence, to accelerate the reaction in the first step of **6** → **7** → **1** and reduce the overall quantity of by-product **10**, **6** was converted to **1** by adding BmeTC^WT^ to the reaction solution of BmeTC^Y167A/D373C^ (Fig. 2d). The yield of **1** was evaluated via a system, in which the total amount of enzymes BmeTC^WT^ and BmeTC^Y167A/D373C^ was the same as that of a single enzyme. The yield of **1** (46.0%) obtained via the new system, comprising BmeTC^WT^ and BmeTC^Y167A/D373C^ was approximately twice that obtained via BmeTC^Y167A/D373C^ alone (21.5%) and approximately 20 times that of BmeTC^D373C^ (2.2%) (Fig. 6a). Since the 2 enzymes can be co-expressed in the yeast *P. pastoris*^17^, the new system should be applicable in vivo (calculated titer: approximately 2 g **1**/L culture medium in the bioreactor).

### Conversion of synthetic 1 into volatile components

**Figure 7:**
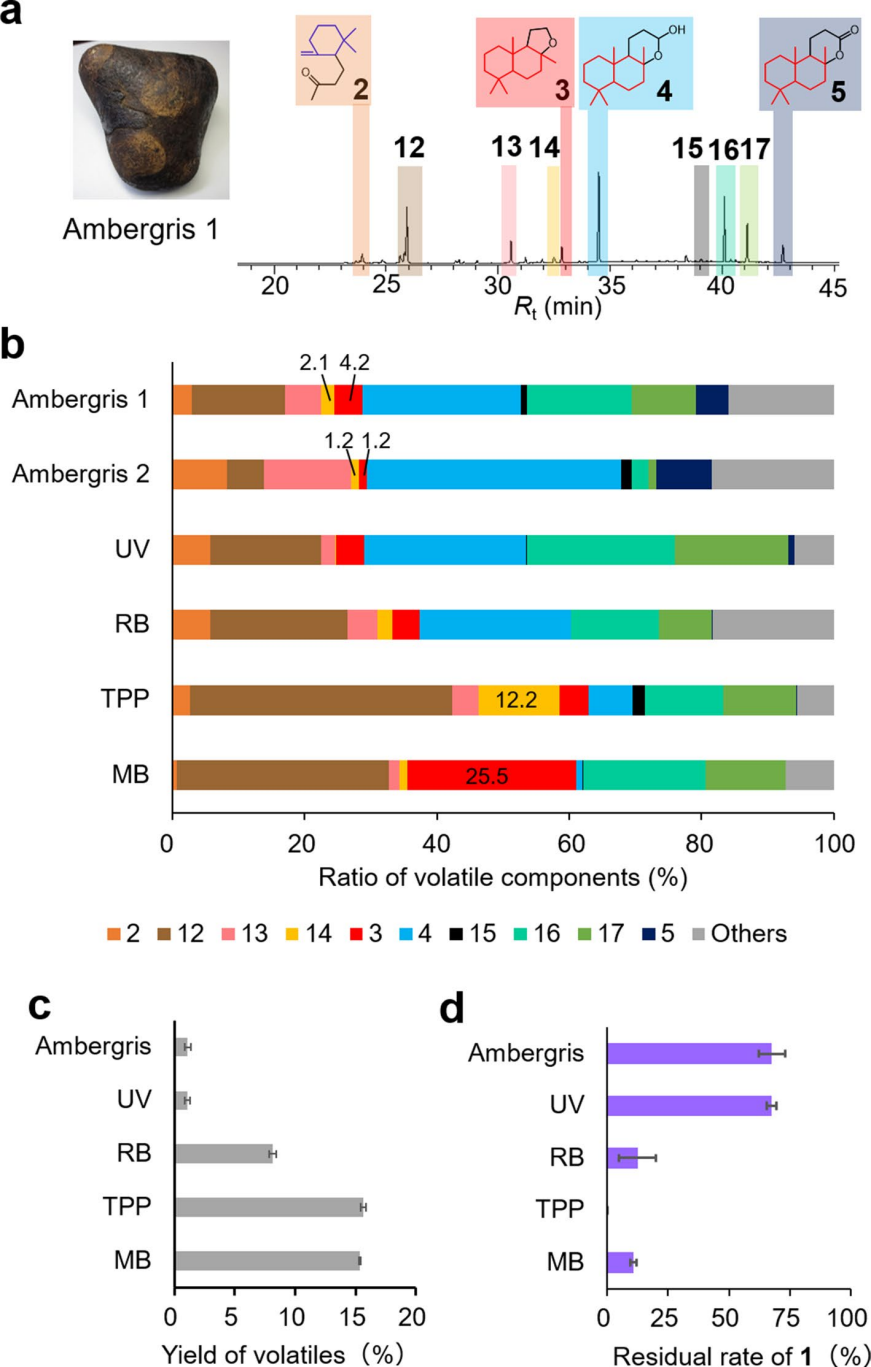
Conversion of **1** into volatile components. (**a**) GC–MS chromatogram of ambergris volatiles. Compounds **2**–**5** were identified, while compounds **12**–**17** were not. (**b**) Percentage of ambergris volatile components and volatile compounds produced by treating **1** with UV light and a photosensitizer (RB, TPP, or MB). The ratio of "others" was calculated using the total amount of compounds, except **2**–**5** and **12**–**17**. (**c**) Yield of ambergris volatile components and volatile compounds produced by treatment of **1** with UV light and a photosensitizer (RB, TPP, or MB). (**d**) Residual rate of **1** in ambergris and reaction solution exposed to UV light and photosensitizer (RB, TPP, or MB).

Enzymatically synthesized **1** was converted to volatiles by ^1^O_2,_ and these volatiles were compared with the volatiles present in ambergris. Ethanol tinctures of two ambergris samples (Supplementary Fig. 7) mainly contain 4 known compounds (**2**–**5**) and 6 other unknown compounds (**12**–**17**) (Fig. 7a,b). The ratio of volatile components was slightly different between the two ambergris samples obtained by us (Fig. 7b), suggesting that the slight difference between their odors could be due to differences in the oxidizing conditions of **1** in the environment. The conversion of **1** to volatile compounds was conducted via UV or visible light treatment using 3 photosensitizers (rose Bengal: RB, 5,10,15,20-tetraphenylporphine: TPP, and methylene blue: MB). Volatile components similar to that of ambergris (**2**–**5** and **12**–**17**) were detected (Fig. 7b and Supplementary Figs. 8–12).

**Figure 8:**
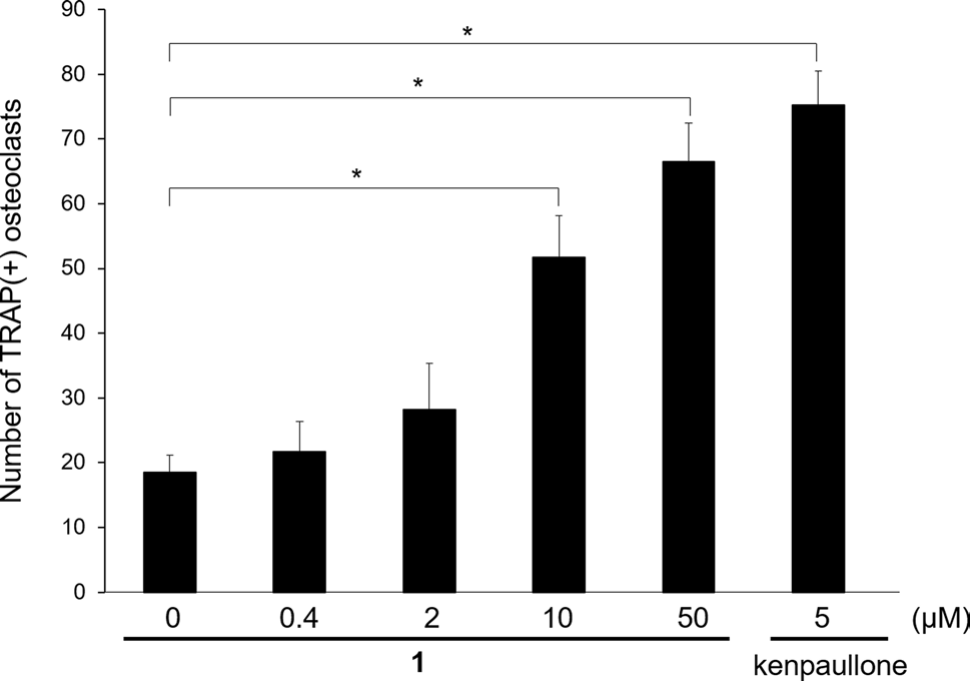
Biological effect of **1** on the osteoclast differentiation. The number of TRAP-positive osteoclasts, differentiated from RAW264.7 cells, was counted in the absence or presence of different concentrations of **1**. Kenpaullone is a positive control. The data are expressed as mean ± S.D. (n = 3, * = P < 0.01).

The yield of volatile components (*ca*. 1%) and the residual rate of **1** (*ca*. 68%) obtained from synthetic **1** via UV treatment were similar to those of ambergris (*ca*. 1% and *ca*. 68%, respectively) (Fig. 7c,d), and also similar to previously reported results obtained by the conversion of **1** to volatile components via visible light using a photosensitizer 5,10,15,20-tetraphenyl-21*H*, 23*H*-porphine copper (II) (yield: *ca*. 1% and residual rate: *ca*. 50%)^11^. In the current study, visible light treatment using 3 photosensitizers (RB, TPP and MB) resulted in higher yields of volatile components (*ca*. 8–15%) and a lower retention of **1** (*ca*. 0–13%) (Fig. 7c,d). The formation rates of volatiles from UV and RB treated **1** were more similar to those of ambergris, while the TPP and MB treated samples had a much higher proportion of **14** (12.2%) and **3** (25.5%), respectively, than the ethanol tinctures of two ambergris samples (1.2–2.1% and 1.2–4.2%) (Fig. 7b). After wetting samples with filter papers and evaporating the solvent, we compared the scents and found that the scents associated with UV and RB treatments of **1** were similar to those obtained from the ethanol tinctures of two ambergris, whereas the scents associated with TPP and MB treatments were different. Notably, the yield of valuable fragrance compound **3**, which is used as a substitute for ambergris samples^1–4^, was 6–21 times higher in MB treated **1** than that obtained from the ethanol tinctures of two ambergris (Fig. 7b). The artificial synthetic system for the major component **1** and odor components of ambergris achieved efficient enzymatic conversion of **6** to **1** as well as efficient conversion of **1** to volatile components by ^1^O_2_.

### Biological activity of 1

Ambergris was previously used as a traditional medicine for various maladies^4,7^. However, the biological activities of natural **1**, the main component of ambergris (*ex.* ambergris samples 1 and 2 contained **1** at *ca*. 68%; Fig. 7d) have not been assessed extensively due to its scarcity. To date, only its aphrodisiac, antinociceptive, and elastase release inhibitory activities are known^5,6,21^. Since the enzymatic synthesis in the current study enabled sufficient production of synthetic **1**, its two biological activities were analyzed. First, we analyzed the effect of **1** on the differentiation of bone cells, osteoblasts and osteoclasts. Extracellular calcium deposited by mature osteoblasts was stained with alizarin red S after cells were incubated with or without 10 μM **1**. However, a significant effect of **1** on the osteoblastic activity was not detected (Supplementary Fig. 13). In contrast, **1** enhanced osteoclastic differentiation at a concentration of 10 μM (Fig. 8). The results indicated that **1** significantly increased the number of mature osteoclasts in a concentration-dependent manner (Fig. 8). The effect of 50 μM **1** was similar to that of 5 μM kenpaullone^22^, which is a strong activator of osteoclastic differentiation. This result suggested that **1** may be a promising drug candidate for osteopetrosis, caused by defective osteoclast function.

**Figure 9:**
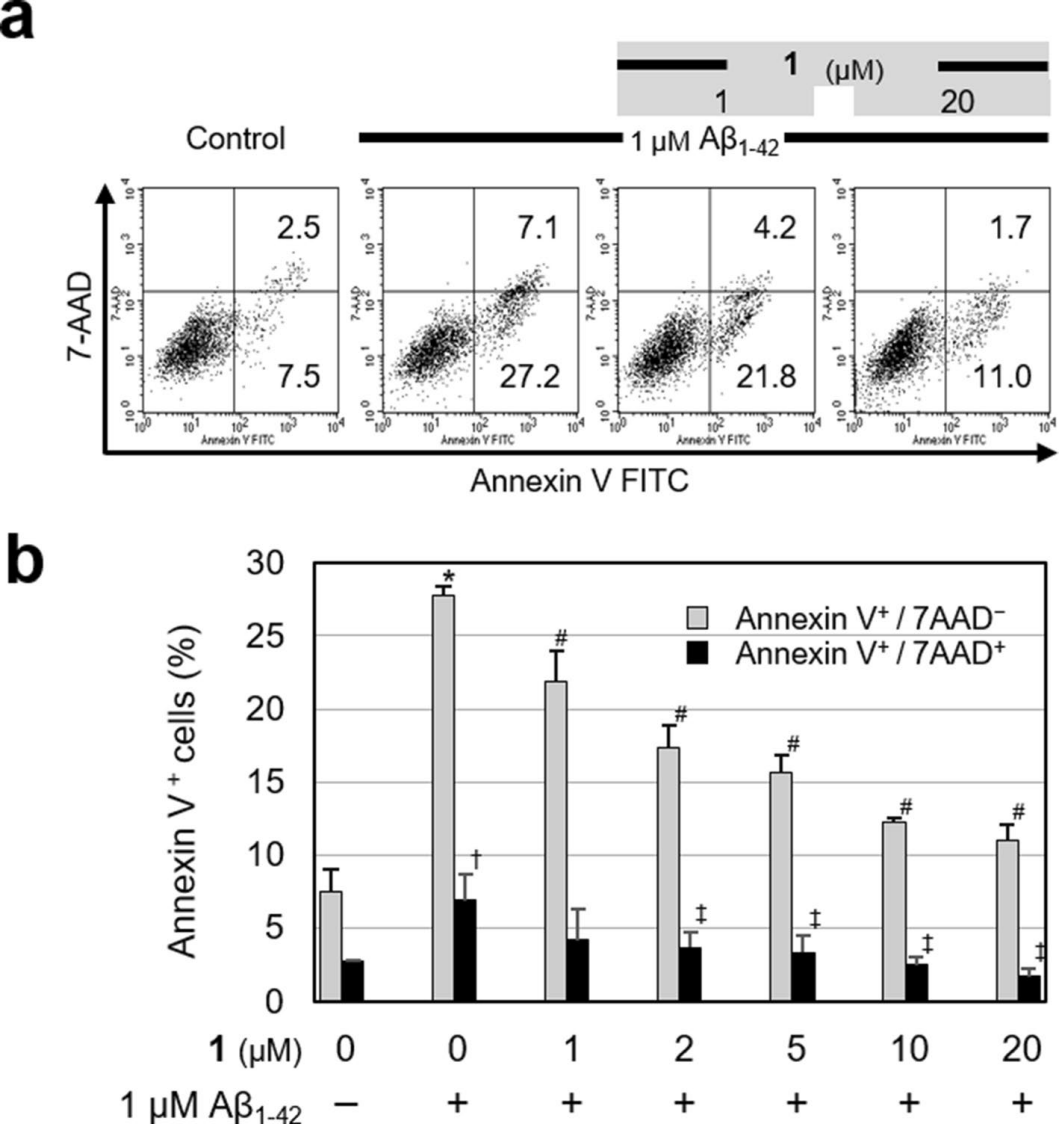
Protective effect of **1** on Aβ_1-42_-induced apoptosis in SK-N-SH cells. Cells were pretreated with different concentrations of **1** (1, 2, 5, 10 and 20 μM) for 24 h before being exposed to 1 μM Aβ_1-42_ for 24 h. Apoptotic cells were analyzed via flow cytometry using an Annexin V/7-AAD staining assay. (**a**) Dot plots of representative experiments. The number in the lower right quadrant signifies the percentage of early apoptotic cells (Annexin V^+^/7-AAD^−^), and the upper right quadrant signifies the percentage of late apoptotic and dead (Annexin V^+^/7-AAD^+^) cells. (**b**) Percentages of early apoptotic cells (Annexin V^+^/7-AAD^−^) and late apoptotic and dead cells (Annexin V^+^/7-AAD^+^). Data are expressed as mean ± SEM; (n = 3). ^*^ and ^†^ refer to the comparison of Aβ_1-42_ alone group versus control group in Annexin V^+^/7-AAD^−^ and Annexin V^+^/7-AAD^+^ cells, respectively; ^*^ or ^†^ = P < 0.01. ^#^ and ^‡^ refer to the comparison of **1** pretreatment groups versus Aβ_1-42_ alone group in Annexin V^+^/7-AAD^−^ and Annexin V^+^/7-AAD^+^ cells, respectively; ^#^ or ^‡^ = P < 0.01.

Next, we analyzed the protective effect of **1** against amyloid β (Aβ)-mediated neurotoxicity. Alzheimer’s disease (AD), the most common type of dementia, is an age-related progressive neurodegenerative disorder characterized by depositions of amyloid β (Aβ), the primary component of senile plaques^23^. Aβ peptides elicit neurotoxicity, leading to neuronal loss and cognitive deficits. We examined the effect of **1** on Aβ-induced apoptotic cell death in human neuroblastoma SK-N-SH cells, in which Aβ_1–42_ was used to induce cell death. Exposure of SK-N-SH cells to 1 µM Aβ_1-42_ for 24 h led to a markedly increased percentage of early apoptotic cells (Annexin V^+^/7-AAD^−^), as well as late apoptotic and dead cells (Annexin V^+^/7-AAD^+^, Fig. 9). Aβ_1-42_-induced apoptosis was significantly inhibited by pretreatment with **1** at concentrations of 1–20 µM for 24 h prior to Aβ_1-42_ exposure (Fig. 9). These results implied that **1** possesses the potential to prevent Aβ neurotoxicity. Presently, we are investigating whether **1** modulates apoptosis signaling pathways and comparing the efficacy of **1** and other Alzheimer's drug candidates targeting Aβ neurotoxicity.

## Discussion

The present study redesigned BmeTC to create a new enzyme named “ambrein (**1**) synthase” (BmeTC^Y167A/D373C^), which displays activity beyond its wild-type function (production of **8** and **9**). We also constructed an efficient in vitro artificial biosynthetic pathway, which can be used for mass production of **1** in vivo. The new two-enzyme system (Fig. 2d) gives approximately 20 times more yield of **1** than the most efficient system currently known (BmeTC^D373C^)^17^ (Fig. 6a) and is expected to produce 2 g **1**/L culture medium in yeast *P. pastoris*. Recently, it was hypothesized that **1** is biosynthesized via the pathway **6** → **7** → **1** in sperm whales^24^. In addition, 2 enzymes are utilized to convert symmetric compounds to asymmetric fern onoceroids and carotenoids^25,26^, via a strategy similar to the one we finally adopted. It is interesting that the pathways adopted by nature are similar to the artificial pathways (Fig. 2d) we have developed.

Although earlier studies on the photooxidation of **1** were aimed at mimicking the production of volatile components of natural ambergris and isolating the volatiles ^9–11^, none of the studies aimed to achieve the efficient conversion of **1** to volatiles. In this study, we were able to obtain a yield of 8–15% (Fig. 7c), which was higher than the yields obtained previously for different purposes^9–11^ and the content in the natural ambergris analyzed by us (Fig. 7c). The synthetic system of volatiles constructed by us could change odor depending on the type of photosensitizers used (Fig. 7b). In the future, a variety of odors may be created by examining various reaction conditions including use of different photosensitizers. In addition, although unknown volatile compounds **12**–**17** were detected in this study (Fig. 7a and Supplementary Fig. 12), their structures could not be determined. New odor compounds may be identified in the future if a large amount of **1** is photooxidized. Further, we identified two biological activities of **1**: promotion of osteoclast differentiation and prevention of Aβ neurotoxicity (Figs. 8 and 9). However, it remains unclear how this compound performs these activities. Identification of an intracellular target molecule of **1** may allow the discovery of therapeutic agents for osteopetrosis and Alzheimer's disease in the future.

This study differed from the conventional biosynthesis studies that have aimed to reconstruct natural biosynthetic pathways. It was a challenge to synthesize a rare natural product (**1**) whose biosynthetic pathway remains unclear, with an artificial biosynthetic route using an enzyme created in the laboratory. Many new natural products have been discovered by genome mining. However, if the biosynthetic enzyme is of a new type or if the natural producer of the product is unknown, genome mining cannot be performed. Therefore, it will be important in the future to synthesize desired compounds by artificially creating new biosynthetic enzymes. In addition, the system constructed in this study can be synthesize **1** analogues and fragrance analogues by redesigning enzymes and using substrate analogues, and will lead to the creation of compounds with numerous odors and biological activities beyond those found in nature in the future.

## Methods

### General

*E. coli* JM109 (Takara, Shiga, Japan) was used for sequencing analysis, and *E. coli* BL21(DE3) (Takara), pColdTF (Takara), and pColdI (Takara) were used to express BmeTC^X^ genes. NMR spectra were recorded using a Bruker DPX 400 spectrometer (Billerica, MA, USA) at 400 MHz for protons (^1^H) and 100 MHz for carbon (^13^C). GC–MS was performed on a JMS-T100GCV spectrometer (JEOL, Tokyo, Japan) equipped with a DB-1 capillary column (30 m × 0.25 mm × 0.25 µm; J&W Scientific. Inc., Folsom, CA, USA), using the EI mode operated at 70 eV. GC analyses were performed using a Shimadzu GC-2014 chromatograph equipped with a flame ionization detector and using a DB-1 capillary column (30 m × 0.25 mm × 0.25 µm; J&W Scientific, Inc.). GC and GC–MS conditions for the BmeTC^X^ products were as follows: injection temperature = 300 °C, column temperature = 220–300 °C (1 °C min^−1^). GC and GC–MS conditions for the volatile compounds were as follows: injection temperature = 200 °C, column temperature = 40–300 °C (5 °C min^−1^) for GC and 30–300 °C (5 °C min^-1^) for GC–MS. Compound **6** was purchased from Wako Pure Chemical Industries, Ltd. (Osaka, Japan). Two ambergris samples (NSMT M55020 and NSMT M55019; Supplementary Fig. 7) stored in the National Museum of Nature and Science (Japan) for more than 30 years were used for the analysis of volatile components.

### Isolation and structural analysis of 1, 7, 10, and 11 synthesized from substrate 6 by BmeTC^***X***^

Compound **11,** biosynthesized in the yeast *P. pastoris,* was identified previously by MS analysis^17^. However, no NMR data were available for **11**. Therefore, isolation and structural analysis of **11** was performed in the present study. Compounds **1**, **7,** and **10** were isolated for use as substrates for enzymatic reactions and material for conversion into volatile components. As a typical example, the method used to isolate **1**, **7**, **10,** and **11** produced by BmeTC^D373C/L596A^ is described below. Compounds **1**, **7,** and **10** were synthesized and isolated via a method similar to BmeTC^D373C/L596A^, mainly using BmeTC^Y167A/D373C^, BmeTC^WT^, and BmeTC^D373C^, respectively.

The expression and preparation of the a cell-free extract was basically the same as in Ref. 20 as described below. *E. coli* BL21(DE3) harboring pColdTF-BmeTC^D373C/L596A^ was grown at 37 °C in LB medium (1 L) with 100 μg mL^-1^ ampicillin^20^. Expression of the recombinant protein was induced by adding 0.1 mM IPTG when OD_600_ reached ~ 0.6^20^. Further cultivation of BL21(DE3) recombinants was performed for 24 h at 15°C^20^. *E. coli* cells expressing recombinant BmeTC^D373C/L596A^ were harvested by centrifugation and resuspended in buffer A (15 mL/5 g) containing 50 mM Tris–HCl (pH 7.5), 2.5 mM dithiothreitol, 1 mM EDTA, 0.1% ascorbic acid and 0.1% Tween-80^20^. Cells were disrupted by sonication with UP200s (Hielscher Ultrasonics GmbH) at 4–10 °C for 15 min^20^. The resulting suspension was centrifuged at 12,300 × g for 20 min (twice)^20^. The pellet was discarded, and the resulting supernatant was used as the cell-free extract.

To isolate product **11** formed by BmeTC^D373C/L596A^, **6** (7.5 mg) was emulsified with Tween 80 (150 mg) in buffer A (75 mL), and incubated with the cell-free extract (300 mL) at 30 °C for 112 h. Subsequently, 15% KOH/MeOH solution (450 mL) was added to the reaction mixture and lipophilic products were extracted with *n*-hexane (400 mL × 3) and concentrated. The gas chromatogram of *n*-hexane extract is shown (Supplementary Fig. 14). The *n*-hexane extract (5.29 g) was partially purified using silica gel (250 g) column chromatography with *n*-hexane and *n*-hexane/EtOAc (100:20). The fraction (Fra. A: 5.0 mg) eluted with *n*-hexane contained substrates **6**, **8**, **10**, and **11**, whereas the fraction (Fra. B: 188.0 mg) eluted with *n*-hexane/EtOAc (100:20) contained **1**, **7**, and **9**. Pure **11** (oil; 1.5 mg) and **10** (oil; 0.1 mg) were obtained by SiO_2_ HPLC (Inertsil 100A, 7.6 × 250 mm; GL Science) with *n*-hexane from Fra. A, and pure **1** (oil; 2.1 mg) and **7** (oil; 0.6 mg) were obtained by SiO_2_ HPLC (Inertsil 100A, 7.6 × 250 mm; GL Science) with *n*-hexane:THF (100:2) from Fra. B.

The structure of compound **11** was determined using MS (Supplementary Fig. 15) and NMR (Supplementary Figs. 16–21). HR-EI-MS detected *m*/*z* 410.3904 [M]^+^ (calculated 410.3913 for C_30_H_50_). The structures and purity (> 99%) of **1**, **7**, and **10** were confirmed by ^1^H NMR to be consistent with those of previous reports^15,27,28^.

### Analysis of BmeTC^***X***^ products using a cell-free system

Construction of pColdTF-BmeTC^WT^ and pColdTF-BmeTC^Y167A^ has been previously reported^20,28^. Site-directed mutagenesis of pColdTF-BmeTC^WT^ and pColdTF-BmeTC^D373C^ was performed using the Quik Change Site-directed Mutagenesis Kit (Agilent Technologies, Santa Clara, CA, USA) and primers which are listed in Supplementary Table 2. The pColdTF-BmeTC^Y257A^ was synthesized by Genewiz (Morrisville, NC, USA) using codon-optimized sequences for *E. coli*. *E. coli* BL21(DE3) harboring pColdTF-BmeTC^X^ (X: WT, D373C/A/F/G/H/L/M/N/Q/S/W, Y167A, Y255A, Y257A, N302A, L596A and F600A) was grown at 37 °C in LB medium (1 L) with 100 μg mL^-1^ ampicillin. The expression and preparation of the a cell-free extract was basically the same as in Ref. 20 as described below. Expression of the recombinant protein was induced by adding 0.1 mM IPTG when OD_600_ reached ~ 0.6. BL21(DE3) recombinants were further cultured for 24 h at 15°C^20^. *E. coli* cells expressing recombinant BmeTC^X^ were harvested by centrifugation and resuspended in buffer A (3 mL/g cells) containing 50 mM Tris–HCl (pH 7.5), 2.5 mM dithiothreitol, 1 mM EDTA, 0.1% ascorbic acid and 0.1% Tween-80^20^. Cells were disrupted via sonication using an UP200s (Hielscher Ultrasonics GmbH) at 4–10 °C for 15 min^20^. The resulting suspension was centrifuged at 12,300 × *g* for 20 min (twice)^20^. The pellet was discarded, and the resulting supernatant was used as cell-free extracts. Sodium dodecyl sulfate polyacrylamide gel electrophoresis (SDS-PAGE) using 10% gel confirmed that all cell-free extracts contained approximately the same amount of BmeTC^X^ (Supplementary Fig. 1).

To analyze BmeTC^X^ products, the substrate (**6**, **7**, or **10**; 0.1 mg) was emulsified with Tween 80 (20 mg) in buffer A (1 mL), and incubated with the cell-free extracts containing BmeTC^X^ (4 mL) at 37 °C for 64 h. After 15% KOH/MeOH solution (6 mL) was added to the reaction mixture, the lipophilic products were extracted with *n*-hexane (10 mL × 3) and concentrated. Next, the *n*-hexane extract containing the products and residual substrate was analyzed by GC and GC–MS. Standard deviations were calculated from the results of 3 replicates.

### Analysis of BmeTC^***X***^ products using purified enzymes

In order to analyze the correct enzyme activity, BmeTC^X^ (X: WT, D373C, Y167A/D373C, Y257A/D373C, N302A/D373C and D373C/L596A) was expressed without the fused TF-tag. The BmeTC^WT^ gene was excised from the *Nde*I and *Xho*I sites of pColdTF-BmeTC^WT^, and introduced into the same site of pColdI to construct pColdI-BmeTC^WT^. Next, pColdI-BmeTC^D373C^ and pColdI-BmeTC^Y167A/D373C^ were synthesized by Genewiz (Morrisville, NC, USA) using codon-optimized sequences for *E. coli,* following which pColdI-BmeTC^Y257A/ D373C^ and pColdI-BmeTC^N302A/ D373C^ were prepared via a Quik Change Site-directed Mutagenesis Kit (Stratagene) using pColdI-BmeTC^D373C^ as a template and the primers listed in Supplementary Table 3. Then pColdI-BmeTC^X^ was introduced into BL21(DE3) together with pGro7, and soluble BmeTC^X^ was expressed. The expression and purification of BmeTC^X^ was basically the same as in Ref. 29 as described below. After culturing as in the case of pColdTF-BmeTC^WT^, cells expressing the recombinant protein were harvested by centrifugation and disrupted by sonication in buffer B [20 mM Tris–HCl (pH 7.9) and 300 mM NaCl] (30 mL/L cultured cells) containing 10 mM imidazole and 0.1% Tween 80 at 4 °C^29^. The homogenate was centrifuged at 18,270 × *g* for 20 min to prepare the supernatant containing soluble His-tagged fusion protein, which was loaded into a Ni–NTA agarose column (0.2 mL; Qiagen, Hilden, Germany), followed by washing with 10 mL of buffer B containing 10 mM imidazole and then by 12 mL of buffer B containing 50 mM imidazole and 0.1% Tween 80^29^. The purified protein was eluted with 3 mL buffer B containing 250 mM imidazole and 0.1% Tween 80, and buffer-exchanged into 3 mL buffer C [50 mM Tris–HCl (pH 7.5), 2.5 mM dithiothreitol, 1 mM EDTA, 300 mM NaCl and 0.1% Tween-80] by gel-filtration chromatography using a Sephadex G-10 column (GE Healthcare, Pittsburgh, PA, USA)^29^. The expression and purification of BmeTC^X^ were analyzed via 10% SDS-PAGE (Supplementary Fig. 5).

The reaction mixture used to analyze the BmeTC^X^ products in buffer C (total volume: 1 mL) contained 8.2 mM (50 μg) substrate (**6**, **7** or **10**) emulsified with 1 mg Tween 80 and 1.4 μM (50 μg) purified BmeTC^X^. Reactions were performed at 37 °C for 64 h. As shown in the representative example in Supplementary Fig. 22, the time-dependent activities of BmeTC^X^ were linear at 64 h. After 15% KOH/MeOH solution (1.2 mL) was added to the reaction mixture, the lipophilic products were extracted using *n*-hexane (2 mL × 3) and the products and the residual substrate was analyzed using GC and GC–MS. Standard deviations were calculated from the results of 3 replicates.

### Conversion of 1 into volatile components

Two ambergris tinctures were prepared with 1 mg ambergris/ 200 µL (95% EtOH). UV treatment of **1** was performed by irradiating a sample [1 mg **1**/200 µL (95% EtOH)] in a glass vial with a UV lamp (15 W) at 26 °C for 6 weeks. Visible light treatment of **1** using a photosensitizer was carried out by irradiating a sample [1 mg **1**/200 µL (RB and MB: 95% EtOH; TPP: dichloromethane)] in a glass vial with LED visible light lamp (60 W) at 26 °C for 4 h, followed by adding the photosensitizer (RB and MB: 100 µM; TPP: 50 µM) every hour and stirring. The samples were directly injected for analysis of volatile compounds by GC and GC–MS. The compounds **2**–**5** were identified by comparing the EIMS spectra of **2**–**5** with those of the NIST library (Supplementary Figs. 8–11). Quantification of volatile compounds by GC was performed by comparison with the peak area of authentic **3** (Kao, Tokyo, Japan). Standard deviations were calculated from the results of 3 replicates.

### Cell culture and treatments

Murine pre-osteoblastic MC3T3-E1 cells (RIKEN Cell Bank, Tsukuba, Japan) were cultured in Minimum Essential Medium Eagle-alpha modification (α-MEM) supplemented with 10% fetal bovine serum (FBS), 100 μg/mL streptomycin, and 100 U/mL penicillin. For induction of osteoblastic differentiation, the cells were incubated in α-MEM complete medium supplemented with 10 mM β-glycerol phosphate, 50 μg/mL ascorbic acid, 10 nM dexamethasone, and 10 μM **1** or 0.1% DMSO as a control; the medium was exchanged with fresh medium every 3 d. Murine macrophage-like pre-osteoclastic RAW264.7 cells (ATCC, Manassas, VA, USA) were cultured in α-MEM medium supplemented with 10% FBS, 2 mM L-glutamine, 100 U/mL penicillin, and 100 μg/mL streptomycin. In order to induce osteoclastic differentiation, 100 ng/mL sRANKL (Oriental Yeast, Japan) was added to the medium with different concentrations of **1** or 0.1% DMSO as a control and incubated for 4 d with 5% CO_2_ at 37 °C. Human neuroblastoma SK-N-SH cells were obtained from Cell Resource Center for Biomedical Research, Institute of Development, Aging and Cancer, Tohoku University (Sendai, Japan) and cultured in DMEM supplemented with 10% FBS, 1 mM sodium pyruvate and 1% penicillin/streptomycin. SK-N-SH cells were seeded at a density of 1 × 10^6^ cells/mL, and following overnight incubation, treated with or without **1** (1–20 μM) for 24 h. Subsequently, culture supernatants containing **1** were removed, and the cells were exposed to 1 µM Aβ_1-42_ (Wako, Osaka, Japan) for an additional 24 h to induce Amyloid β (Aβ)-mediated neurotoxicity.

### Alizarin red S staining

MC3T3-E1 cells cultured in the osteoblastic differentiation medium for 3 weeks and were subsequently fixed with 4% PFA for 30 min at 24 °C. Finally, cells were incubated for 45 min at 24 °C with 1% alizarin red S (FUJIFILM Wako Pure Chemical Corp., Osaka, Japan), and then washed by distilled water^30^.

### TRAP staining

Matured osteoclasts were fixed with 10% glutaraldehyde for 15 min at 37 °C and subsequently incubated for 10 min at 37 °C in TRAP staining buffer containing 10 mg/mL naphthol AS-MX phosphate, 0.3 mg/mL Fast Red Violet LB Salt, 0.1 M sodium acetate, 0.3 M potassium tartrate, 0.1% Triton X-100, and 0.1 M acetic acid^31^. TRAP-positive osteoclasts with more than 3 nuclei were considered as mature osteoclasts and counted using a light microscope (Olympus IX73, Tokyo, Japan).

### Apoptosis assay by flow cytometry

For the apoptosis assay, flow cytometric analysis was performed using FITC-Annexin V (Biolegend, San Diego, CA, USA) and 7-Amino-Actinomycin (7-AAD; Biolegend). Cells were pretreated with or without **1** for 24 h, followed by 24 h exposure to 1 µM Aβ_1-42_. Subsequently, cells were harvested, washed twice with phosphate-buffered saline containing 0.1% bovine serum albumin, and resuspended in Annexin V binding buffer at 1 × 10^6^ cells/mL. Thereafter, cells were stained with FITC-Annexin V (5 µg/mL) and 7-AAD (0.5 μg/mL) for 15 min in the dark. Finally, cells were analyzed using FACSCalibur flow cytometry system (BD Bioscience, San Jose, CA, USA). A minimum of 10,000 events was collected and the percentage of apoptotic cells was calculated using the CellQuest software (BD Bioscience).

### Statistical analysis

Statistical significance was determined by one-way analysis of variance followed by the Tukey–Kramer test for multiple comparisons at P < 0.01.

## Supplementary information

Supplementary Information.

## Data Availability

Data supporting the findings of this study are available within the article and the Supplementary Information files, and from the corresponding author upon reasonable request.
