# Supplementary material for: Construction of an artificial system for ambrein biosynthesis and investigation of some biological activities of ambrein
Source: Sci Rep. 2020 Nov 12;10:19643. doi: 10.1038/s41598-020-76624-y (PMC7661701; doi:10.1038/s41598-020-76624-y)
Supplement: Supplementary file 1 — Supplementary Information. [file 41598_2020_76624_MOESM1_ESM.pdf]

Supplementary Information

Construction of an artificial system for ambrein biosynthesis and investigation of some biological activities of ambrein

Yota Yamabe[1#], Yukina Kawagoe[1#], Kotone Okuno[1], Mao Inoue[1], Kanako Chikaoka[1], Daijiro Ueda[1], Yuko Tajima[2], Tadasu K Yamada[2], , Yoshito Kakihara[3]*, Takashi Hara[1]* and Tsutomu Sato[1]*

1 Department of Agriculture, Faculty of Agriculture, and Graduate School of Science and Technology, Niigata University, 8050 Ikarashi-2, Nishi-ku, Niigata, Japan
2 Department of Zoology, National Museum of Nature and Science, Tsukuba, Ibaraki, Japan
3 Division of Dental Pharmacology, Faculty of Dentistry & Department of Tissue Regeneration and Reconstruction, Niigata University Graduate School of Medical and Dental Sciences, 2-5274 Gakkocho-dori, Chuo-ku, Niigata, 951-8514, Japan.

#These authors contributed equally to this work.

*Correspondence.
E-mail: kakihara@dent.niigata-u.ac.jp
E-mail: harata@agr.niigata-u.ac.jp
E-mail: satot@agr.niigata-u.ac.jp

Contents

Figures	Page
**Supplementary Fig. 1.** SDS-PAGE of cell-free extracts of *E. coli* containing recombinant BmeTC^{X}.	S3
**Supplementary Fig. 2.** Ratio of onoceroids and "others" shown in Fig. 5a (cell-free + **6**).	S4
**Supplementary Fig. 3.** Ratio of onoceroids and "others" shown in Fig. 5b (cell-free + **7**).	S5
**Supplementary Fig. 4.** Ratio of "others" shown in Fig. 5c (cell-free + **10**).	S6
**Supplementary Fig. 5.** SDS-PAGE of purified BmeTC^{X}.	S7
**Supplementary Fig. 6.** Gas chromatogram of the products formed by the purified enzymes.	S8
**Supplementary Fig. 7.** The two ambergris samples used in this study.	S9
**Supplementary Fig. 8.** Identification of **2** by EI-MS.	S10
**Supplementary Fig. 9.** Identification of **3** by EI-MS.	S11
**Supplementary Fig. 10.** Identification of **4** by EI-MS.	S12
**Supplementary Fig. 11.** Identification of **5** by EI-MS.	S13
**Supplementary Fig. 12.** EI-MS spectrum of **12-17** contained in ambergris tincture.	S14
**Supplementary Fig. 13.** Effects of the biological activity of **1** on the differentiation of osteoblasts.	S15
**Supplementary Fig. 14.** Gas chromatogram of *n*-hexane extract of reaction mixture in which BmeTC$^{D373C/L596A}$ was incubated with **6**.	S16
**Supplementary Fig. 15.** EI-mass spectrum of **11**.	S17
**Supplementary Fig. 16.** NMR assignment of **11** measured in C_6D_6.	S18
**Supplementary Fig. 17.** ^{1}H NMR spectrum of **11** measured in C_6D_6.	S19
**Supplementary Fig. 18.** ^{13}C NMR spectrum of **11** measured in C_6D_6.	S20
**Supplementary Fig. 19.** ^{1}H-^{1}H COSY spectrum of **11** measured in C_6D_6.	S21
**Supplementary Fig. 20.** HSQC spectrum of **11** measured in C_6D_6.	S22
**Supplementary Fig. 21.** HMBC spectrum of **11** measured in C_6D_6.	S23
**Supplementary Fig. 22.** Time-dependent activities of BmeTC^{WT} (a) and BmeTC$^{Y167A/D373C}$ (b).	S24
**Tables**	
**Supplementary Table 1.** Amount of **1** (mg L^{-1}) produced by in vivo systems.	S25
**Supplementary Table 2.** Sequences of primers to introduce mutations into pColdTF-BmeTC^{WT}.	S26
**Supplementary Table 3.** Sequences of primers to introduce mutations into pColdI-BmeTC^{D373C}.	S27

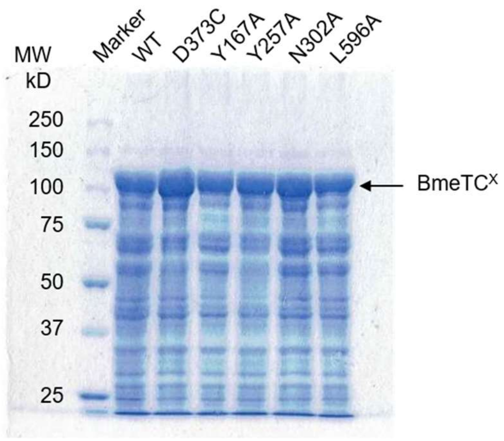

Supplementary Fig. 1. SDS-PAGE of cell-free extracts of *E. coli* containing recombinant BmeTC^X.

As a representative example, 6 enzymes (WT and 5 variants) are shown, and approximately the same amount as WT was confirmed for all the enzymes described in this study. X: WT, D373C, Y167A, Y257A, N302A and L596A.

a

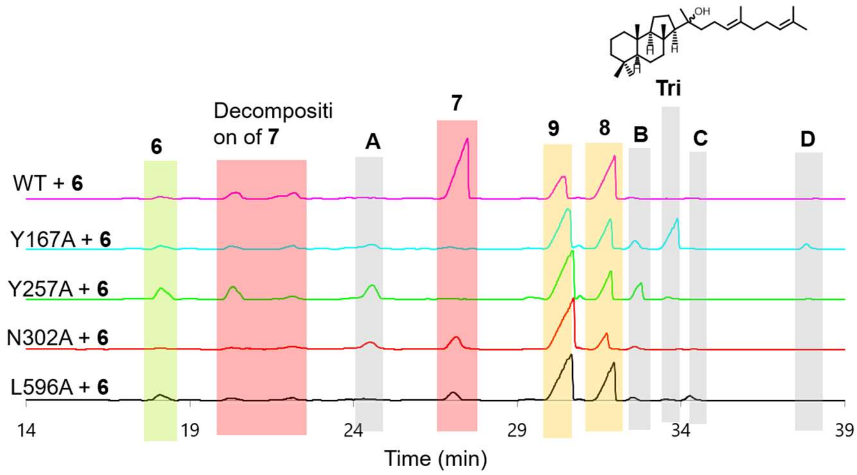

b

			Onoceroids		Others				
	**6**	**7**	**9**	**8**	**Tri**	**A**	**B**	**C**	**D**
**WT**	1.7	44.3	18.1	35.9	-	-	-	-	-
**Y167A**	3.0	1.5	40.6	20.2	21.0	6.1	4.7	-	2.9
**Y255A**	-	52.8	23.6	23.6	-	-	-	-	-
**Y257A**	9.6	-	51.9	15.8	-	14.6	8.2	-	-
**N302A**	-	13.4	67.1	10.1	-	7.5	1.9	-	-
**L596A**	4.7	7.0	50.0	30.1	-	-	2.0	6.3	-
**F600A**	4.5	62.5	12.7	11.7	-	8.5	-	-	-

Supplementary Fig. 2. Ratio of onoceroids and "others" shown in Fig. 5a (cell-free + **6**).
(a) Gas chromatogram of reaction products and residual substrate. "Tri" has been identified as a tricyclic compound as depicted above (Ref. 20) while the others (A-D) have not been identified.
(b) Ratio of reaction products and residual substrate.

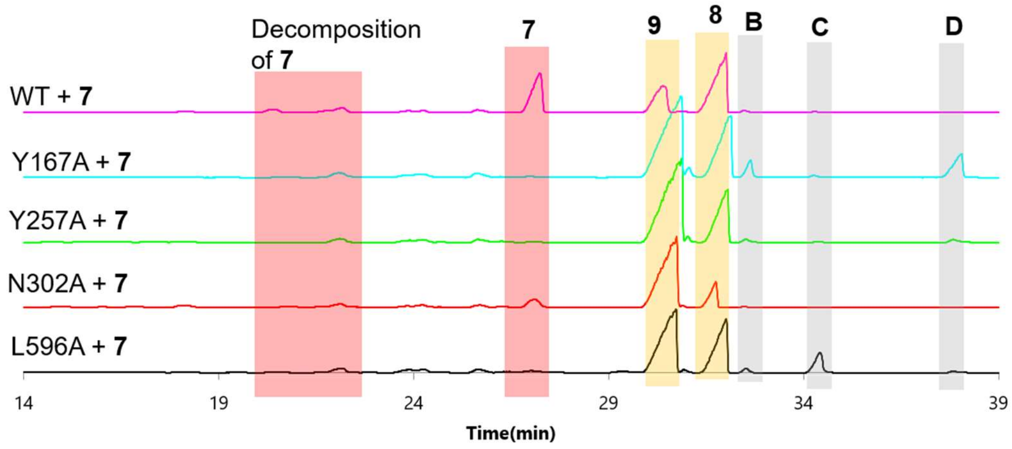

	Onoceroids			Others		
	7	9	8	B	C	D
**WT**	25.5	25.0	48.1	1.3	-	-
**Y167A**	-	58.6	31.4	2.0	-	7.9
**Y255A**	37.0	29.6	33.4	-	-	-
**Y257A**	-	70.3	27.9	1.0	-	0.8
**N302A**	6.9	79.7	13.4	-	-	-
**L596A**	-	56.5	33.8	1.7	7.4	0.6
**F600A**	36.6	34.3	28.3	0.8	-	-

Supplementary Fig. 3. Ratio of onoceroids and “others” shown in Fig. 5b (cell-free + **7**).
(a) Gas chromatogram of reaction products and residual substrate. B-D have not been identified.
(b) Ratio of reaction products and residual substrate.

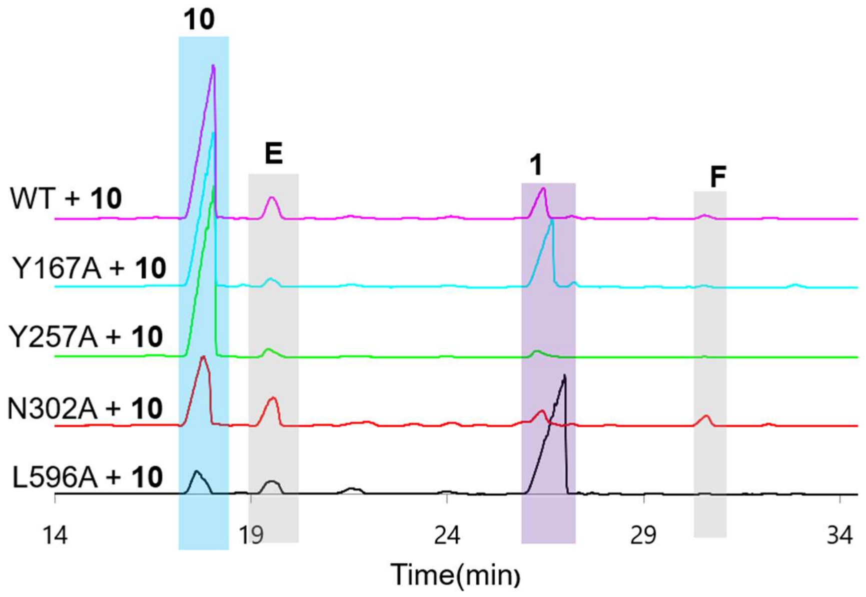

			Others	
	**10**	**1**	**E**	**F**
**WT**	81.0	10.7	8.3	-
**Y167A**	70.0	27.0	3.0	-
**Y255A**	81.7	12.8	5.5	-
**Y257A**	93.6	2.9	3.5	-
**N302A**	60.2	13.3	20.9	5.6
**L596A**	14.7	78.5	6.8	-
**F600A**	91.0	2.8	3.6	2.5

Supplementary Fig. 4. Ratio of “others” shown in Fig. 5c (cell-free + **10**).

(a) Gas chromatogram of reaction products and residual substrate. E and F have not been identified.

(b) Ratio of reaction products and residual substrate.

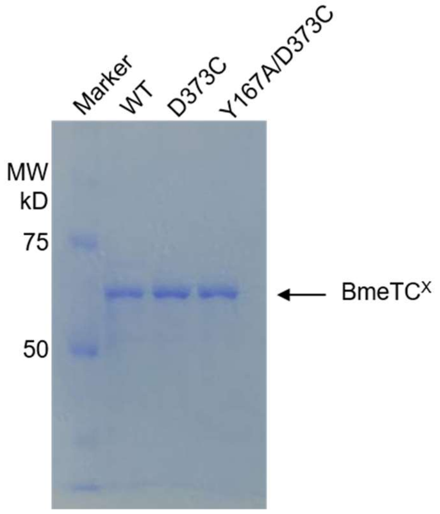

Supplementary Fig. 5. SDS-PAGE of purified BmeTC^X.

As a representative example, 3 enzymes (WT and two variants) are shown. All other enzymes used in this study were also confirmed to have similar purity. X: WT, D373C, Y167A/D373C.

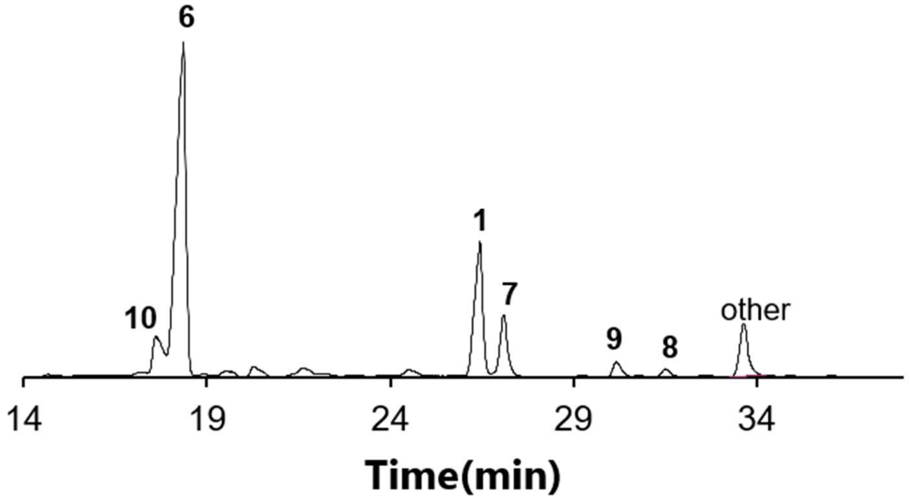

Supplementary Fig. 6. Gas chromatogram of the products formed by the purified enzymes. As a representative example, the result of BmeTC$^{Y167A/D373C}$ using substrate **6** is shown. The "other" is the tricyclic compound shown in Supplementary Fig. 2 (Ref. 20).

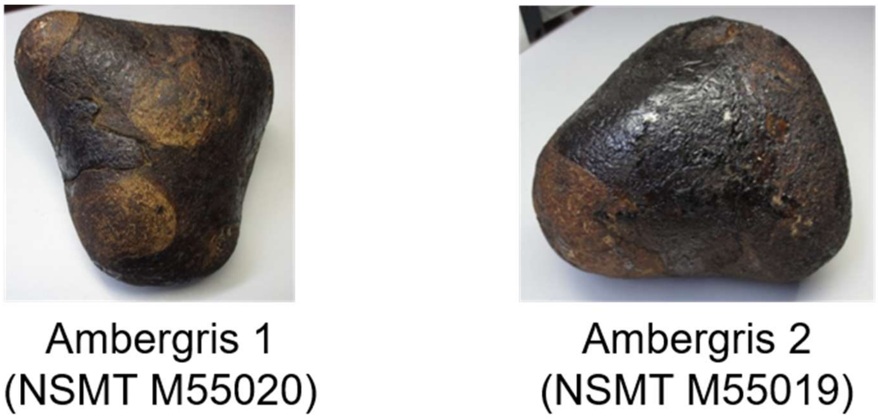

Supplementary Fig. 7. The two ambergris samples used in this study.

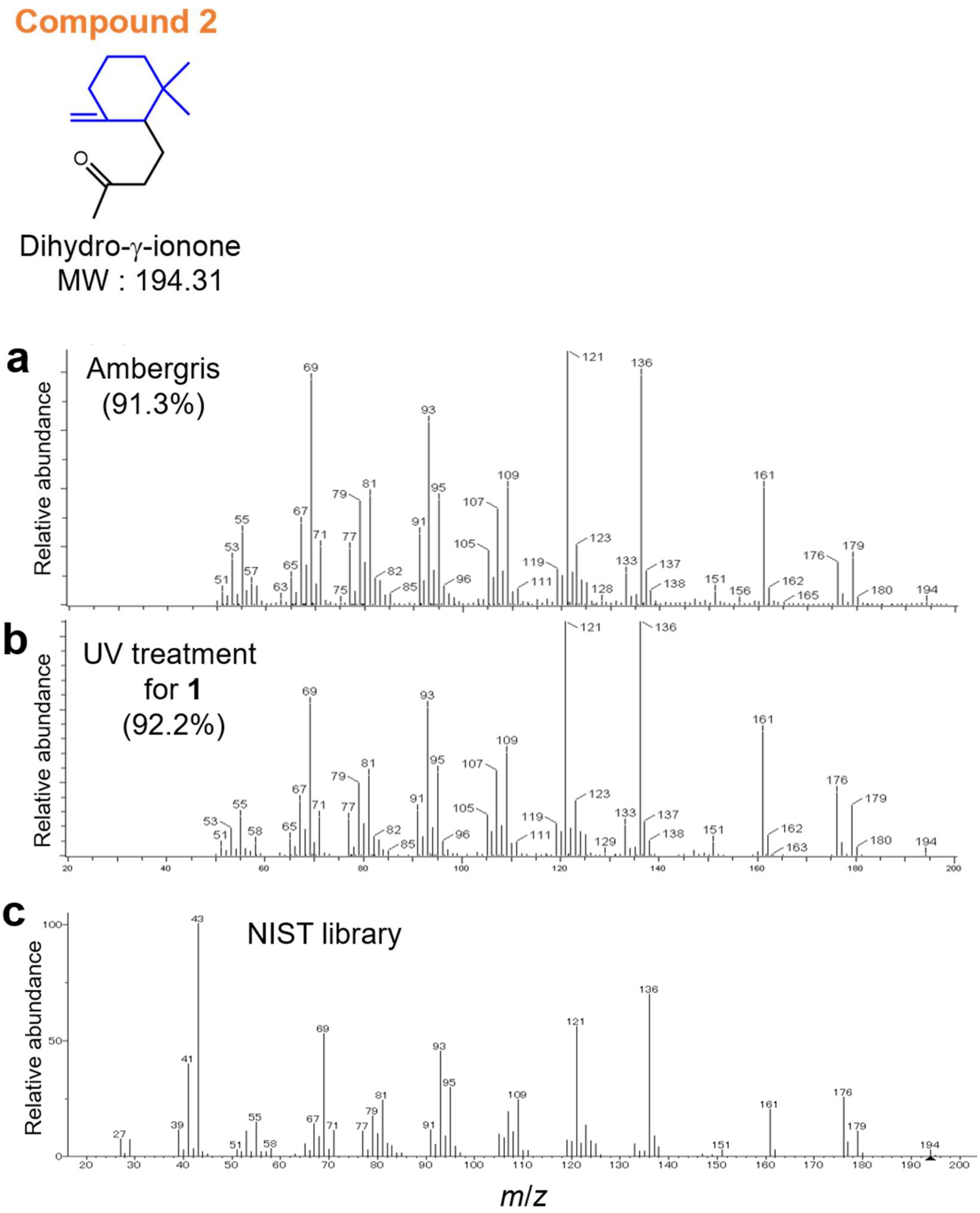

Supplementary Fig. 8. Identification of **2** by EI-MS (R_t = 23.0 min)

(a) EI-MS spectrum of **2** contained in ambergris tincture

(b) EI-MS spectrum of **2** formed by UV treatment of **1**. Similar spectra were obtained from the treatment of **1** with three separate photosensitizers.

(c) NIST library of **2**.

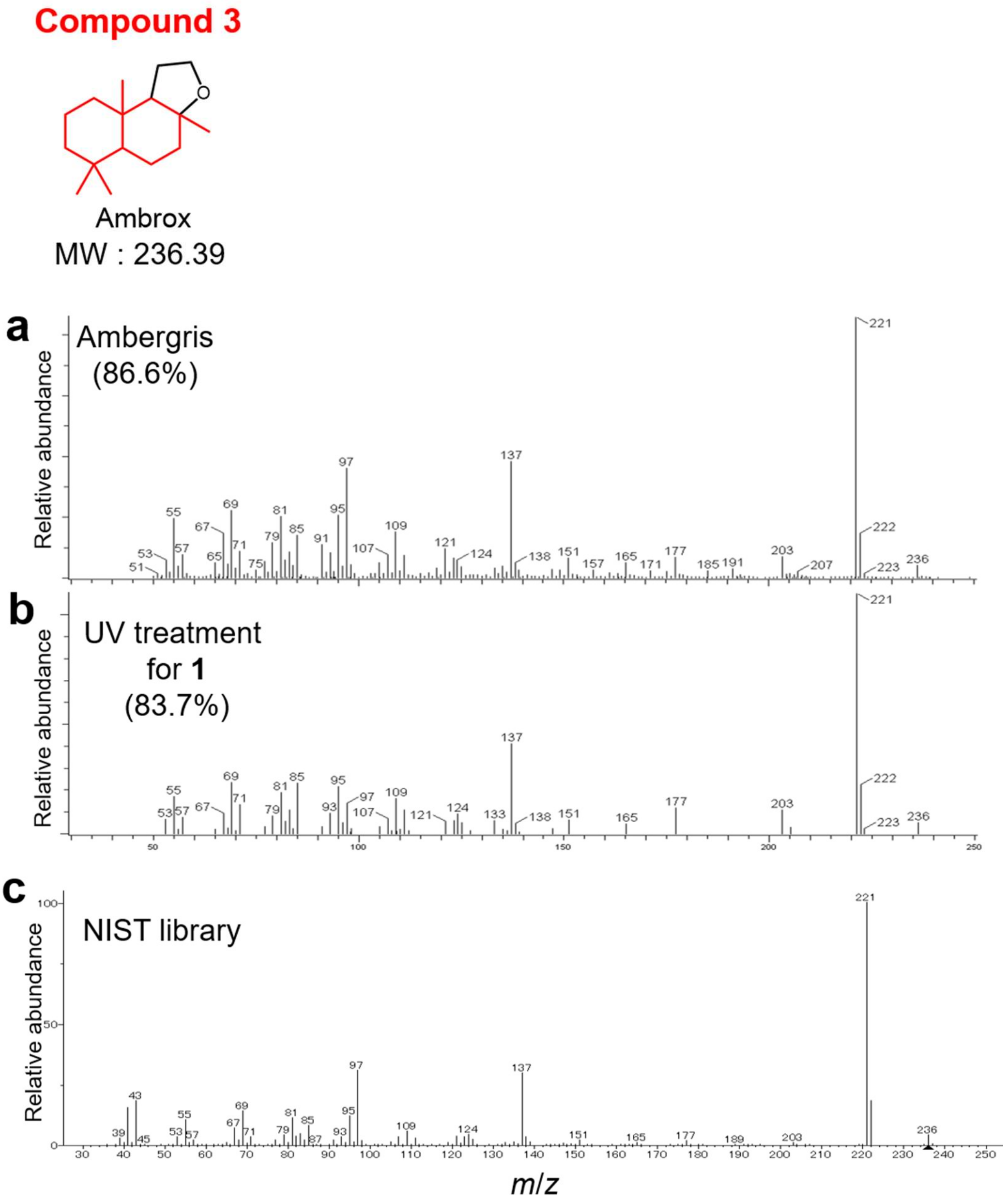

Supplementary Fig. 9. Identification of **3** by EI-MS (R_t = 31.8 min).
(a) EI-MS spectrum of **3** contained in ambergris tincture
(b) EI-MS spectrum of **3** formed by UV treatment of **1**. Similar spectra were obtained from the treatment of **1** with three separate photosensitizers.
(c) NIST library of **3**.

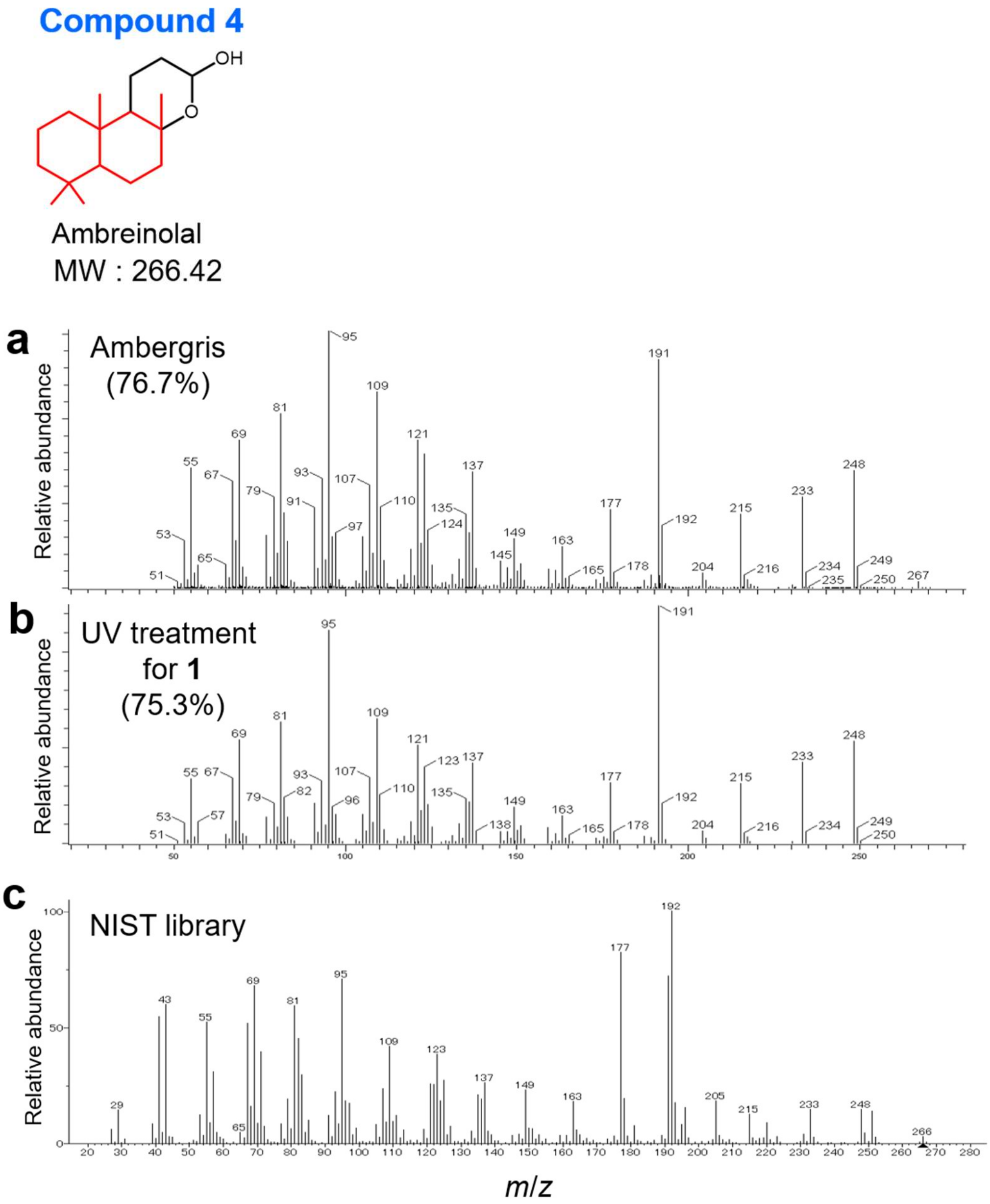

Supplementary Fig. 10. Identification of **4** by EI-MS (R_t = 34.4 min).

(a) EI-MS spectrum of **4** contained in ambergris tincture

(b) EI-MS spectrum of **4** formed by UV treatment of **1**. Similar spectra were obtained from the treatment of **1** with three separate photosensitizers.

(c) NIST library of **4**.

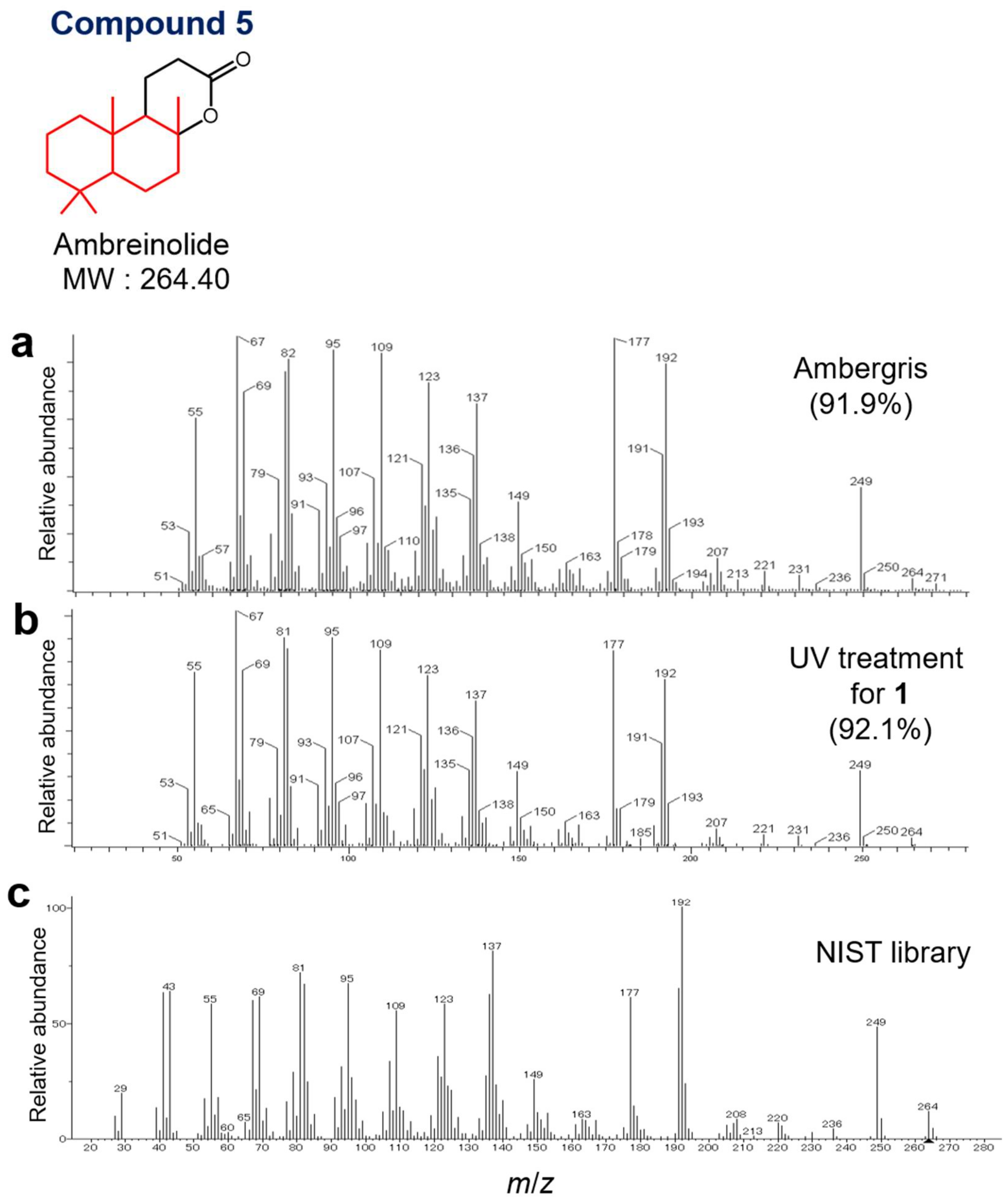

Supplementary Fig. 11. Identification of **5** by EI-MS (R_t = 41.5 min).

(a) EI-MS spectrum of **5** contained in ambergris tincture

(b) EI-MS spectrum of **5** formed by UV treatment of **1**. Similar spectra were obtained from the treatment of **1** with three separate photosensitizers.

(c) NIST library of **5**.

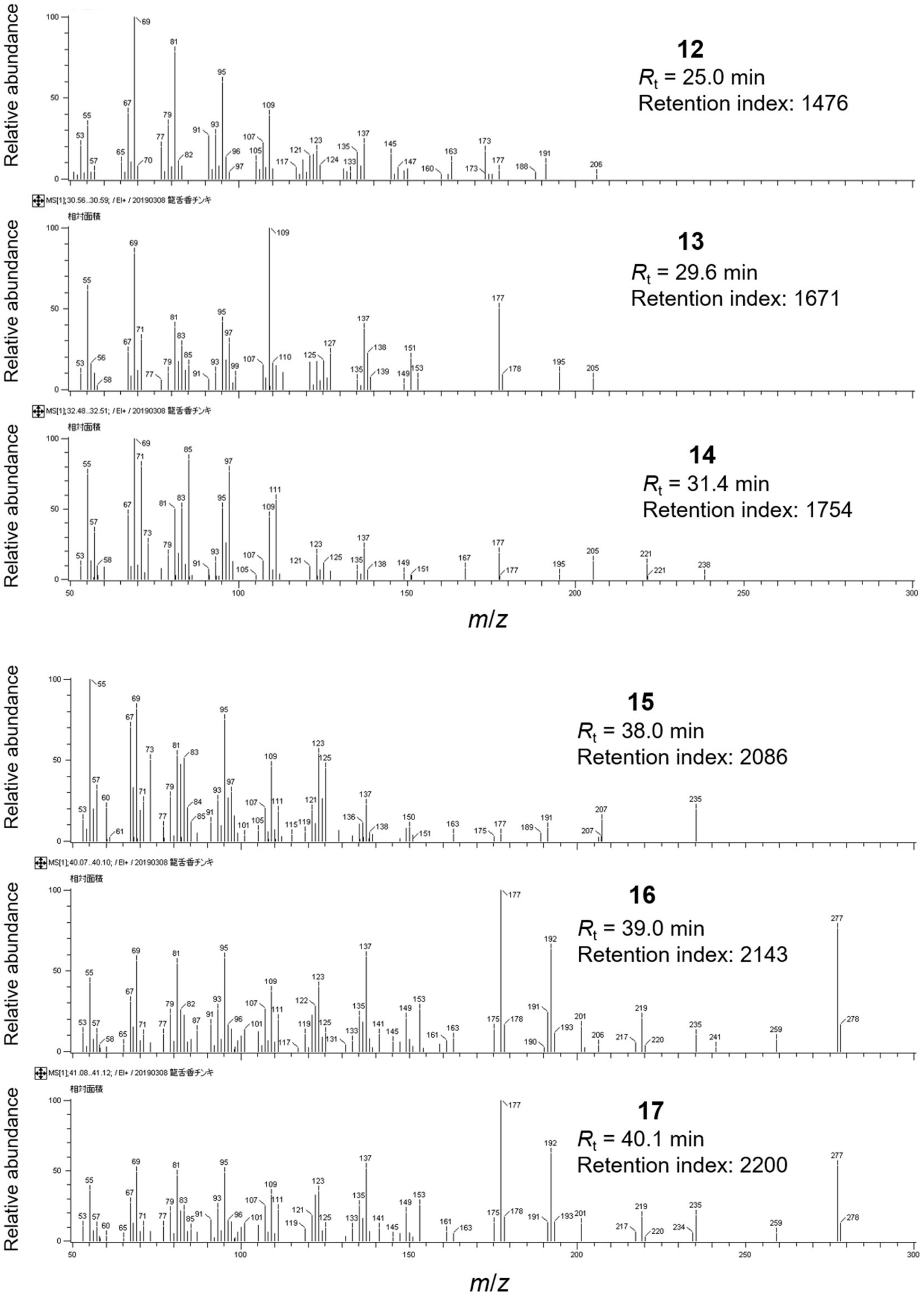

Supplementary Fig. 12. EI-MS spectrum of **12-17** present in ambergris tincture.

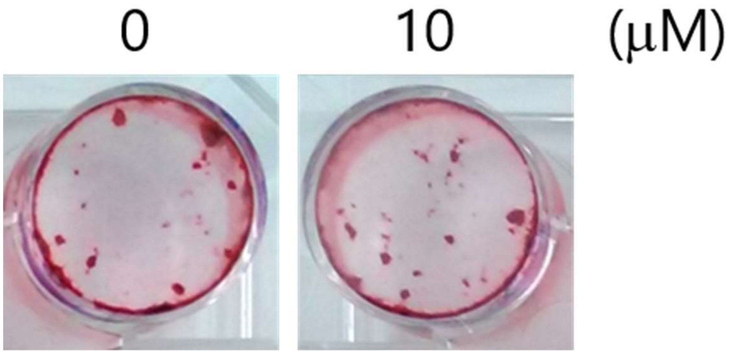

Supplementary Fig. 13. Effects of the biological activity of **1** on the differentiation of osteoblasts.

Alizarin red S staining of osteoblasts differentiated from MC3T3-E1 in the presence of 10 μM **1**.

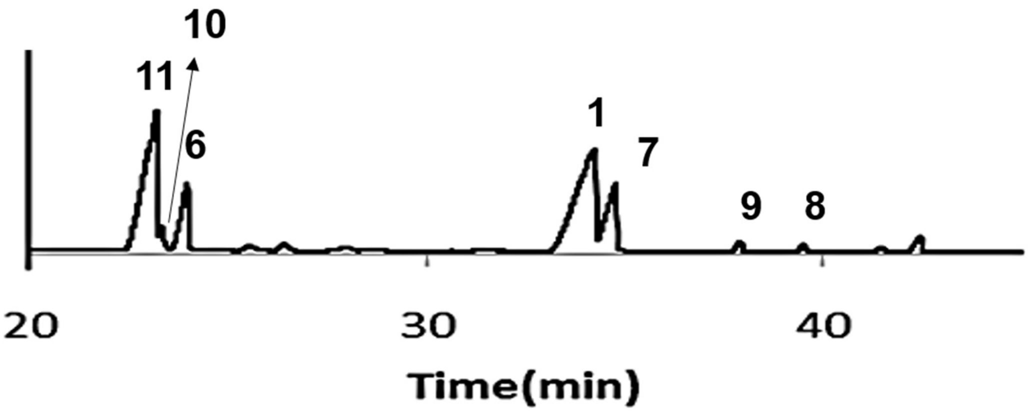

Supplementary Fig. 14. Gas chromatogram of *n*-hexane extract of reaction mixture in which BmeTC$^{D373C/L596A}$ was incubated with **6**.

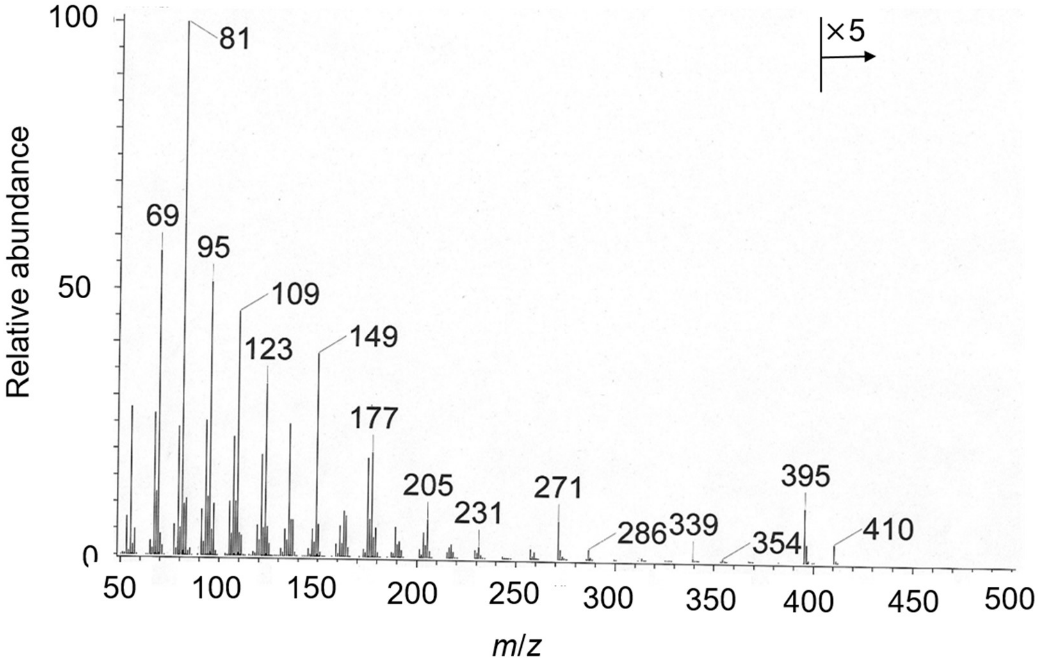

Supplementary Fig. 15. EI-mass spectrum of **11**.

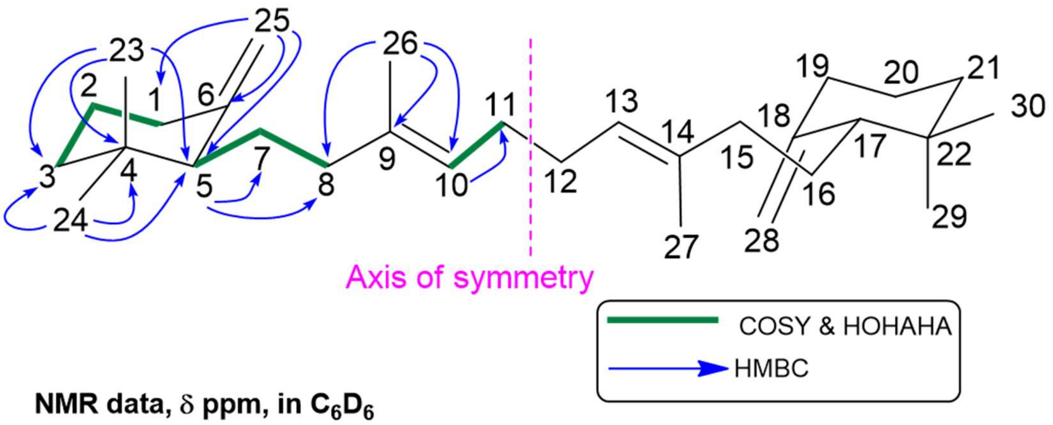

NMR data, δ ppm, in C_6D_6

NO.	^1H	^{13}C	NO.	^1H	^{13}C
1, 19	2.14 (m)	32.7 (t)	9, 14	—	135.8 (s)
2, 20	1.59 (m)	24.1 (t)	10, 13	5.50 (1H, s)	124.6 (d)
3, 21	1.26 (m); 1.52 (m)	36.5 (t)	11, 12	2.30 (m)	28.8 (t)
4, 22	—	35.0 (s)	23, 29	1.08 (3H, s)	28.6 (q)
5, 17	1.87 (m)	53.9 (d)	24, 30	0.98 (3H, s)	26.5 (q)
6, 18	—	149.4 (s)	25, 28	4.83 (1H, s); 5.00 (1H, s)	109.4 (t)
7, 16	1.65 (m); 1.76 (m)	25.2 (t)	26, 27	1.77 (3H, s)	16.3 (q)
8, 15	2.03 (m); 2.26 (m)	38.7 (t)			

Supplementary Fig. 16. NMR assignment of **11** measured in C_6D_6.

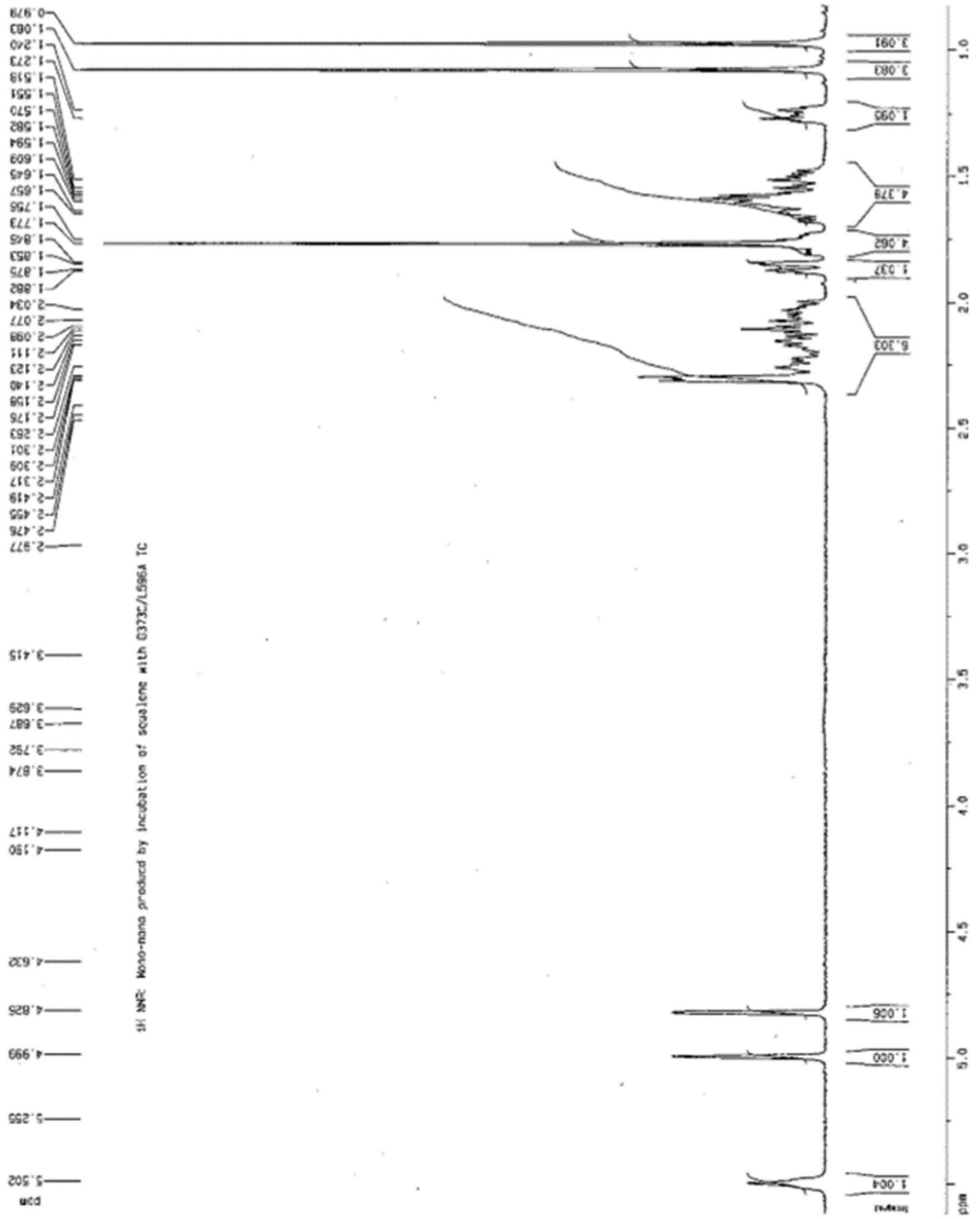

Supplementary Fig. 17. ^{1}H NMR spectrum of **11** measured in C_6D_6.

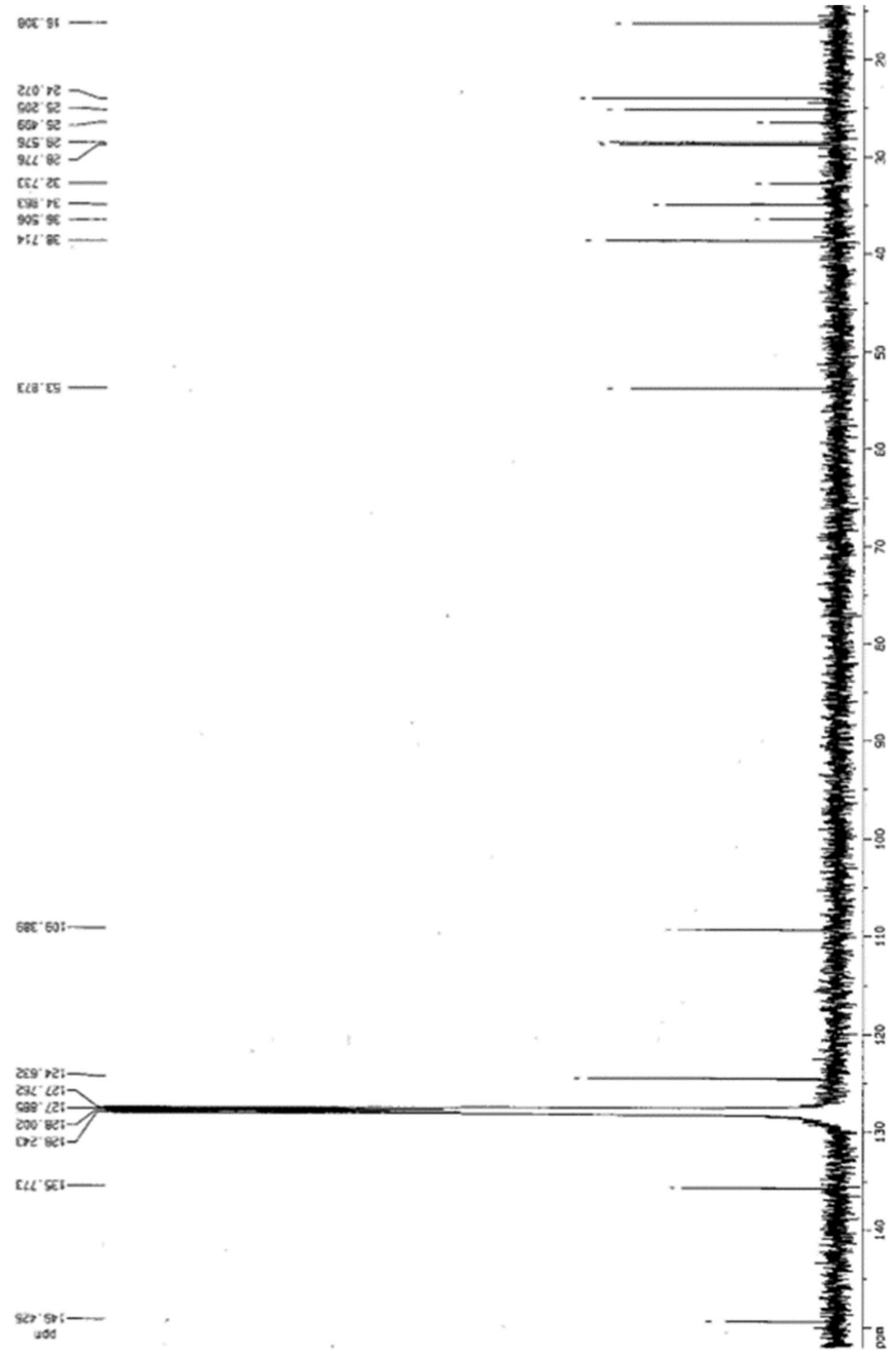

Supplementary Fig. 18. ^{13}C NMR spectrum of **11** measured in C_6D_6.

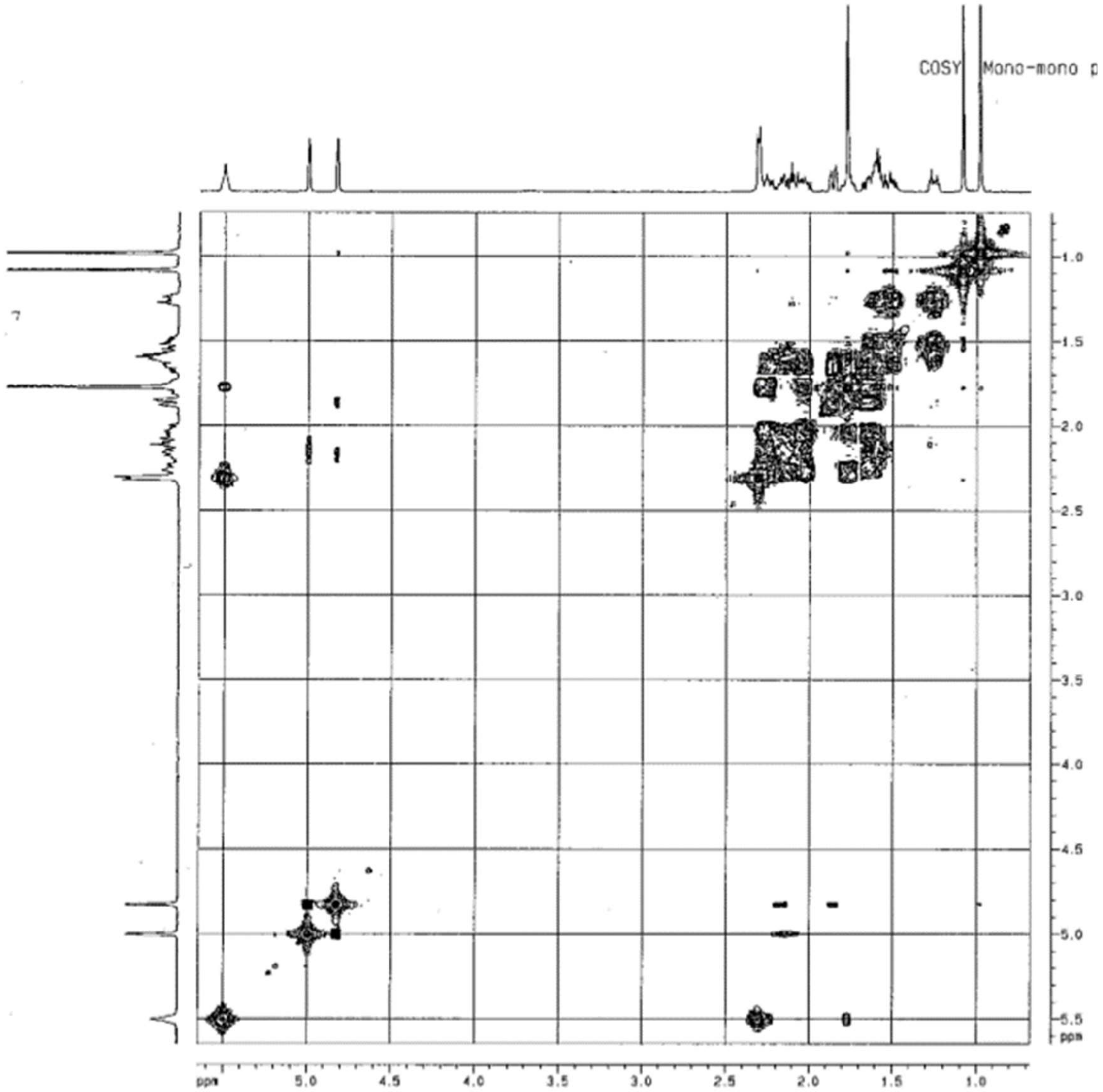

Supplementary Fig. 19. ^1H-^1H COSY spectrum of **11** measured in C_6D_6.

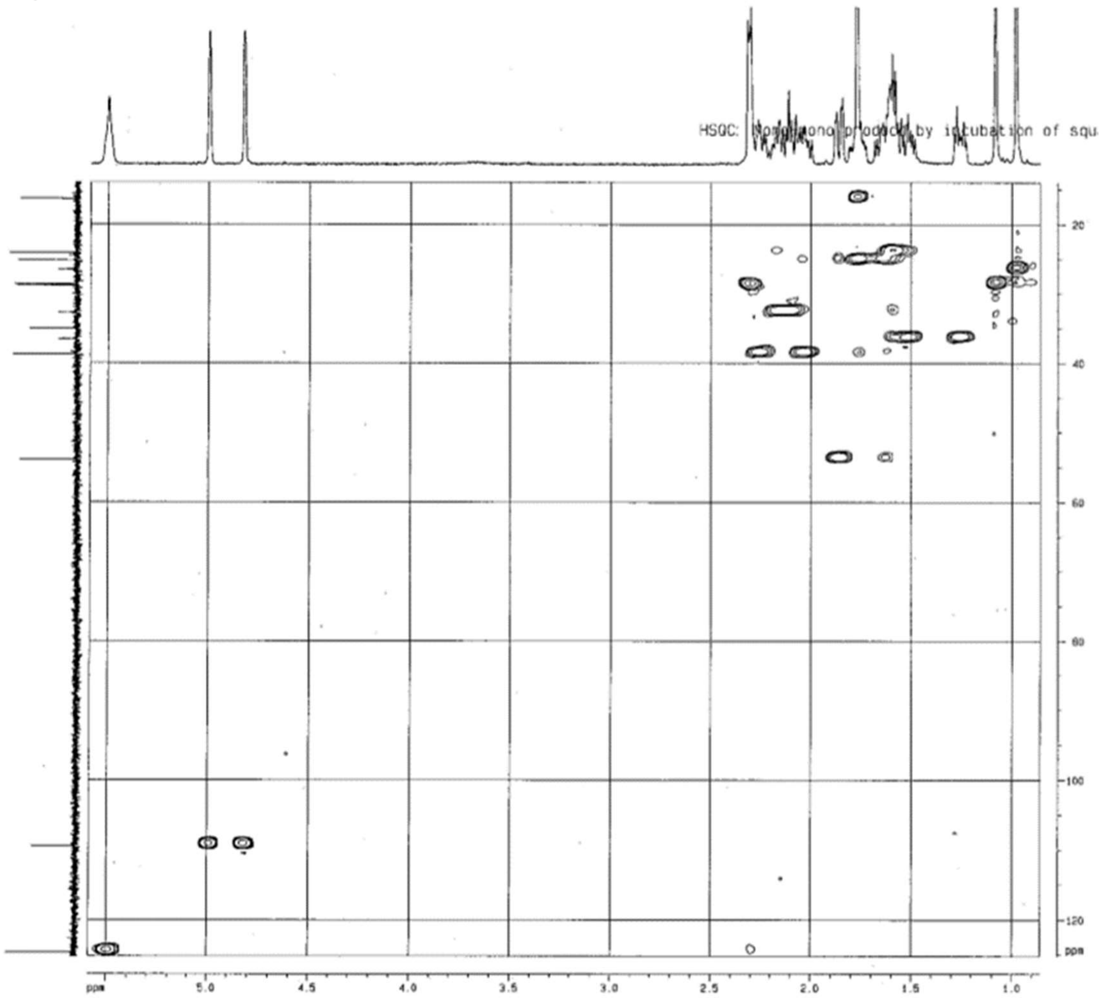

Supplementary Fig. 20. HSQC spectrum of **11** measured in C_6D_6.

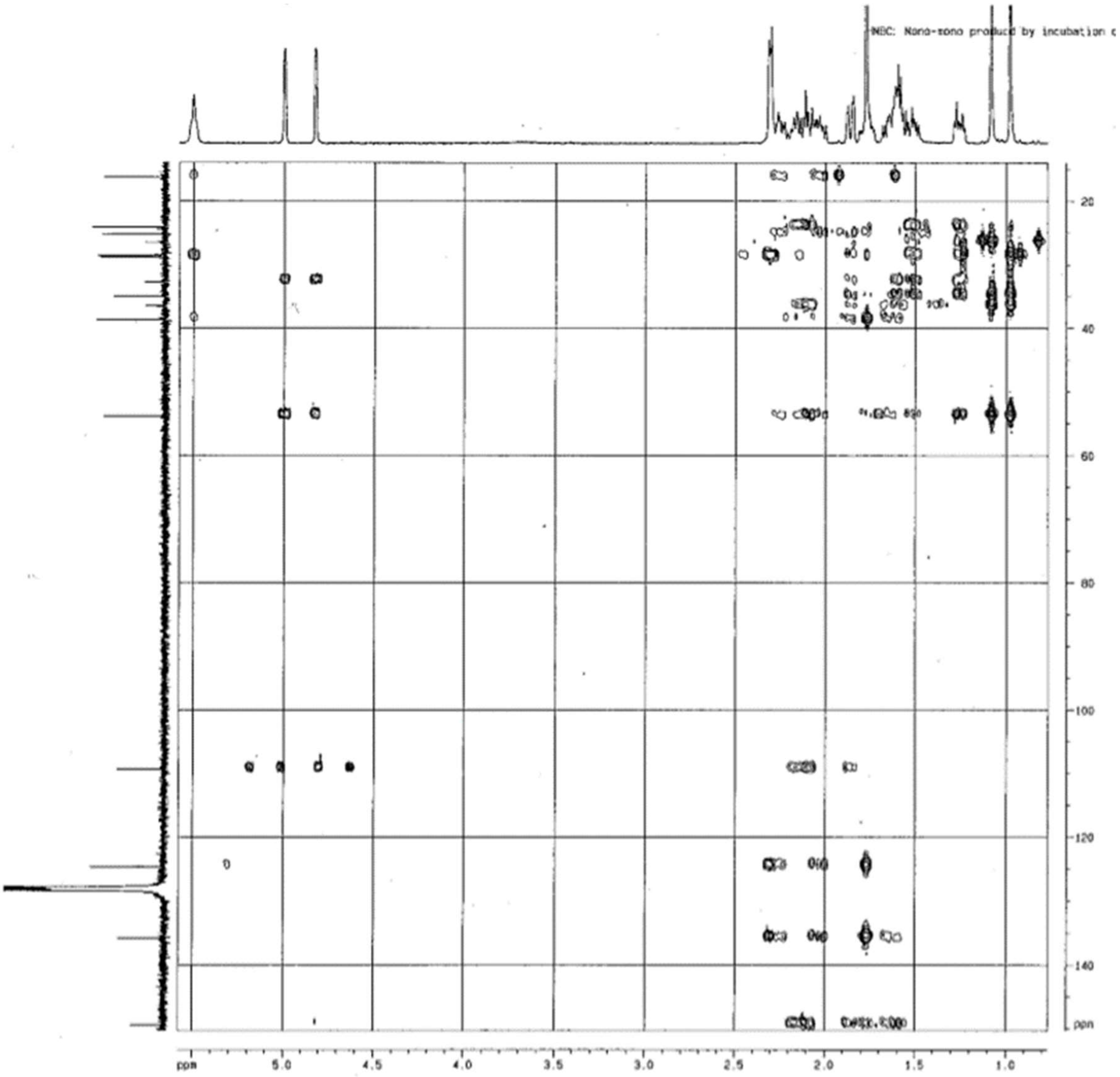

Supplementary Fig. 21. HMBC spectrum of **11** measured in C_6D_6.

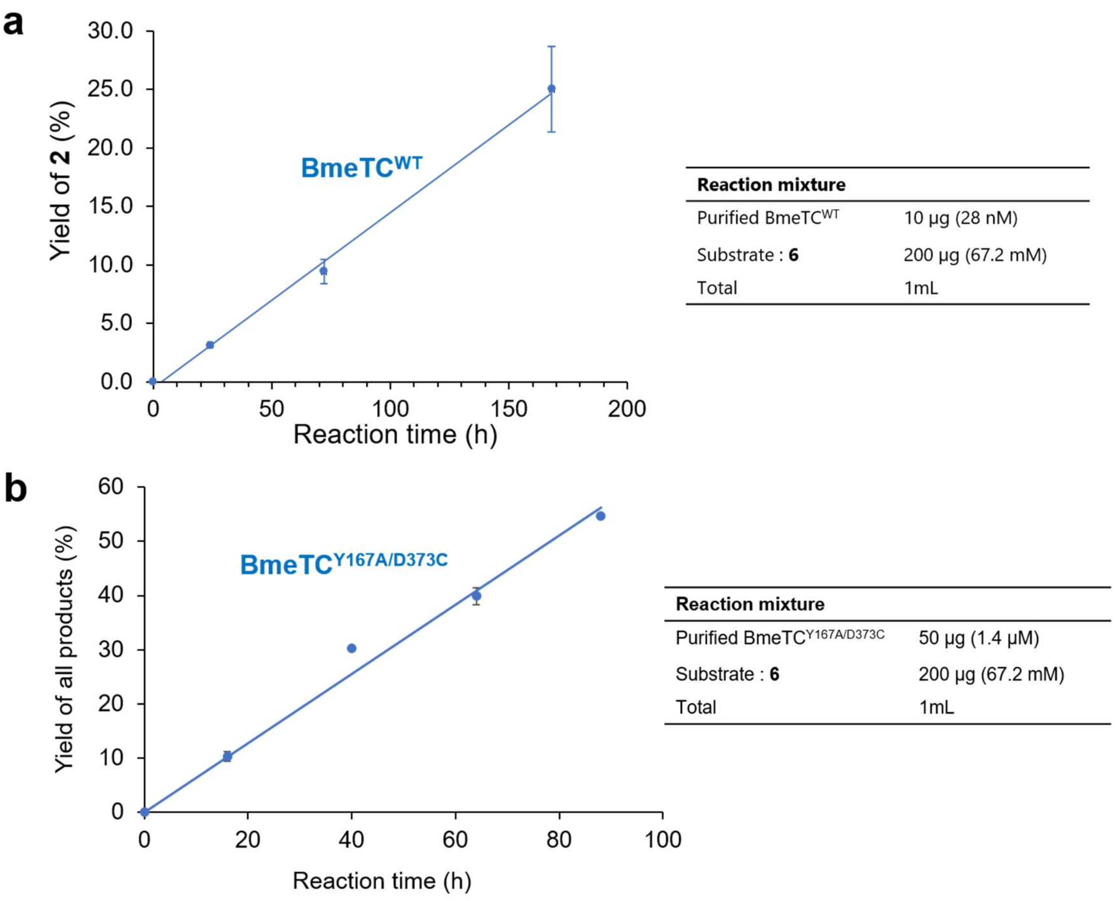

a

Reaction mixture	
Purified BmeTC^{WT}	10 μg (28 nM)
Substrate : **6**	200 μg (67.2 mM)
Total	1mL

b

Reaction mixture	
Purified BmeTC$^{Y167A/D373C}$	50 μg (1.4 μM)
Substrate : **6**	200 μg (67.2 mM)
Total	1mL

Supplementary Fig. 22. Time-dependent activities of BmeTC^{WT} **(a)** and BmeTC$^{Y167A/D373C}$ **(b)**.

To maintain excess substrate, higher amounts of substrate and/or lower amounts of purified enzymes were used than those used under conditions shown in the results (Fig. 6).

Supplementary Table 1. Amount of **1** (mg L^{-1}) produced by *in vivo* systems.

Host	Enzyme	Culture equipment	Amount of **1** (mg L^{-1})	Ref.
Escherichia coli	SHC^{D377C} + $BmeTC^{WT}$	shake flasks	2.6	19
Pichia pastoris	SHC^{D377C} + $BmeTC^{WT}$	shake flasks	1.9	17
Pichia pastoris	$BmeTC^{D373C}$	shake flasks	14.9	17
Pichia pastoris	$BmeTC^{D373C}$	bioreactor	105	17
Saccharomyces cerevisiae	SHC^{D377C} + $BmeTC^{WT}$	shake flasks	0	18
Saccharomyces cerevisiae	$BmeTC^{D373C}$	shake flasks	2.9	18

Supplementary Table 2. Sequence of primers used to introduce mutations into pColdTF-BmeTC^{WT}.

Primer name		Sequence
D373A	F	5’-CCCTGACTTAGACGCTACGTCTGCTGCTATCAGAGC- 3’
	R	5’-GCTCTGATAGCAGCAGACGTAGCGTCTAAGTCAGGG- 3’
D373G	F	5’-CCCTGACTTAGACGGTACGTCTGCTGCTATCAGAGC- 3’
	R	5’-GCTCTGATAGCAGCAGACGTACCGTCTAAGTCAGGG- 3’
D373L	F	5’-CCCTGACTTAGACCTTACGTCTGCTGCTATCAGAGCAC- 3’
	R	5’-GTGCTCTGATAGCAGCAGACGTAAGGTCTAAGTCAGGG- 3’
D373F	F	5’-CCCTGACTTAGACTTTACGTCTGCTGCTATCAGAGCAC- 3’
	R	5’-GTGCTCTGATAGCAGCAGACGTAAAGTCTAAGTCAGGG- 3’
D373W	F	5’-CCCTGACTTAGACTGGACGTCTGCTGCTATCAGAGCAC- 3’
	R	5’-GTGCTCTGATAGCAGCAGACGTCCAGTCTAAGTCAGGG- 3’
D373H	F	5’-CCCTGACTTAGACCATACGTCTGCTGCTATCAGAGC- 3’
	R	5’-GCTCTGATAGCAGCAGACGTATGGTCTAAGTCAGGG- 3’
D373M	F	5’-CCCTGACTTAGACATGACGTCTGCTGCTATCAGAGCTC-3’
	R	5’-GAGCTCTGATAGCAGCAGACGTCATGTCTAAGTCAGGG-3’
D373S	F	5’-CCCTGACTTAGACTCTACGTCTGCTGCTATCAGAGCTC-3’
	R	5’-GAGCTCTGATAGCAGCAGACGTAGAGTCTAAGTCAGGG-3’
D373N	F	5’-CCCTGACTTAGACAATACGTCTGCTGCTATCAGAGCTC-3’
	R	5’-GAGCTCTGATAGCAGCAGACGTATTGTCTAAGTCAGGG-3’
D373Q	F	5’-CCCTGACTTAGACCAGACGTCTGCTGCTATCAGAGCTC-3’
	R	5’-GAGCTCTGATAGCAGCAGACGTCTGGTCTAAGTCAGGG-3’
D373C	F	5’-CAAATAACCCTGACTTAGACTGTACGTCTGCTGCTATCAGAG-3’
	R	5’-CTCTGATAGCAGCAGACGTACAGTCTAAGTCAGGGTTATTTG-3’
Y255A	F	5’- GATGGAACACTCCGCAGCTACGCCACCTCTAC-3’
	R	5’- GTAGAGGTGGCGTAGCTGCGGAGTGTTCCATC-3’
N302A	F	5’-CCACGTGCACGTAGAAGCCTCCACGTCAACCG-3’
	R	5’-CGGTTGACGTGGAGGCTTCTACGTGCACGTGG-3’
L596A	F	5’-CCCACGGGAATTGGTGCTCCTGGACAATTTTATATTCAG -3’
	R	5’- CTGAATATAAAATTGTCCAGGAGCACCAATTCCCGTGGG-3’
F600A	F	5’-GGTCTTCCTGGACAAGCTTATATTCAGTACCACAGCTAC-3’
	R	5’-GTAGCTGTGGTACTGAATATAAGCTTGTCCAGGAAGACC-3’

Supplementary Table 3. Sequences of primers used to introduce mutations into pColdI-BmeTC^{D373C}.

Primer name		Sequence
OP-Y257A	F	5’-GGACGGCACACTGTACAGTGCCGCCACCAGCACCTTTTTTATG-3’
	R	5’-CATAAAAAAGGTGCTGGTGGCGGCACTGTACAGTGTGCCGTCC-3’
OP-N302A	F	5’-GTTGAAAGCAGTACCAGCACCGTTTGG-3’
	R	5’-GGTACTGGCTTCAACGTGCACGTGACC-3’
